# Validation of a patient safety checklist for radiological procedures in hemodynamics

**DOI:** 10.1590/0034-7167-2021-0011

**Published:** 2022-05-11

**Authors:** Flávia Ana Pacheco, Márcia Marques dos Santos Felix, Patrícia da Silva Pires, Elizabeth Barichello, Ana Lúcia De Mattia, Maria Helena Barbosa

**Affiliations:** I Universidade Federal do Triângulo Mineiro. Uberaba, Minas Gerais, Brazil.; II Universidade Federal da Bahia. Vitória da Conquista, Bahia, Brazil.; III Universidade Federal de Minas Gerais. Belo Horizonte, Minas Gerais, Brazil.

**Keywords:** Radiology, Interventional, Validation Study, Patient Safety, Minimally Invasive Surgical Procedures, Checklist, Radiología Intervencionista, Estudios de Validación, Seguridad del Paciente, Procedimientos Quirúrgicos Mínimamente Invasivos, Lista de Verificación, Radiologia Intervencionista, Estudos de Validação, Segurança do Paciente, Procedimentos Cirúrgicos Minimamente Invasivos, Lista de Checagem

## Abstract

**Objectives:**

to carry out cultural adaptation and validation of WHO Surgical Safety Checklist: for Radiological Interventions ONLY to Brazilian Portuguese.

**Methods:**

a methodological research with the following stages of the cultural adaptation process: translation of the instrument, achievement of a consensus in Portuguese, evaluation by a committee of judges, back-translation, achievement of a consensus in English, comparison with the original version, and a pre-test. The psychometric properties of the adapted version were evaluated through interobserver reliability.

**Results:**

the values of the kappa coefficient ranged from moderate to almost perfect in most instrument items, demonstrating that the instrument items were understandable and reliable when applied to the observed context.

**Conclusions:**

the cultural adaptation and validation of face and content of the instrument met the criteria of equivalence between the original and the translated instrument. The tool proved to be understandable and feasible and can be applied in invasive radiological procedures in Brazil.

## RESUMO

**Objetivos:**

realizar adaptação cultural e validação do WHO *Surgical Safety Checklist: for Radiological Interventions ONLY* para o português brasileiro.

**Métodos:**

pesquisa metodológica cujo processo de adaptação cultural percorreu as seguintes etapas: tradução do instrumento, obtenção do consenso em português, avaliação por comitê de juízes, retrotradução, obtenção do consenso em inglês, comparação com a versão original e pré-teste. As propriedades psicométricas da versão adaptada foram avaliadas por meio de confiabilidade interobservadores.

**Resultados:**

os valores do coeficiente Kappa variaram de moderado a quase perfeito na maioria dos itens do instrumento, demonstrando que os itens do instrumento foram compreensíveis e confiáveis quando aplicados ao contexto observado. **Conclusões:** a adaptação cultural e a validação de face e conteúdo do instrumento satisfizeram os critérios de equivalência entre o instrumento original e o traduzido. O instrumento mostrou-se compreensível e viável, podendo ser aplicado em procedimentos radiológicos invasivos no Brasil.

## RESUMEN

**Objetivos:**

realizar adaptación cultural y validación del WHO *Surgical Safety Checklist: for Radiological Interventions ONLY* al portugués brasileño.

**Métodos:**

investigación metodológica cuyo proceso de adaptación cultural recorrió las siguientes etapas: traducción del instrumento, obtención del consenso en portugués, evaluación por comité de jueces, retrotraducción, obtención del consenso en inglés, comparación con la versión original y pretest. Las propriedades psicométricas de la versión adaptada fueron evaluadas por medio de confiabilidad interobservadores.

**Resultados:**

los valores del coeficiente Kappa variaron de moderado a casi perfecto en la mayoría de los ítems del instrumento, demostrando que los ítems del instrumento fueron comprensibles y confiables cuando aplicados al contexto observado.

**Conclusiones:**

la adaptación cultural y la validación de Face y contenido del instrumento satisficieron los criterios de equivalencia entre el instrumento original y el traducido. El instrumento se mostró comprensible y viable, pudiendo ser aplicado en procedimientos radiológicos invasivos en Brasil.

## INTRODUCTION

Patient safety is a constant concern in hospital units with discussions around the world^(1)^. In the United States, the estimate is that 251 thousand deaths occur annually due to complications arising from care errors, which represents 9.5% of deaths in the country and is the third largest cause of mortality, behind only cardiovascular diseases and cancer^(2-3)^. In Brazil, although still underreported, deaths due to care errors are a reality and represent 0.6% of the total adverse events reported^(4)^.

The World Health Organization (WHO) published, in 2009, initiatives to promote patient safety in surgical procedures. Its campaign “Safe Surgery Saves Lives” introduced the concept of a checklist, the Surgical Safety Checklist (SSC), intended to identify and control risks during the three phases of the surgical procedure: before induction of anesthesia, before incision of the skin, and before leaving the operating room^(5)^.

Interventional Radiology is a specialty with a lower incidence of complications and morbidity compared to surgical procedures due to its minimally invasive nature^(6)^. However, invasive radiological procedures have many aspects in common with surgical procedures (complexity, rapid resolution, urgency and emergency, teamwork, etc.) and, accordingly, entail potential risk of failures and complications. Thus, implementing a checklist in interventional radiology can have the same efficacy in patient safety as the surgical checklists^(7)^.

The National Patient Safety Agency (NPSA) has published guidelines for radiologists in implementing the safe surgery requirement^(8)^, and the Royal College of Radiologists (RCR) adapted the checklist of Safe Surgery from the World Health Organization for a checklist specific used in radiological interventions in England and Wales, entitled WHO Surgical Safety Checklist: for Radiological Interventions ONLY^(9)^. Adherence to the checklist as part of a culture of safety by the team is essential. NPSA and RCR advise and encourage its adaptation to meet local needs^(8-9)^.

In Brazil, the surgical checklist is an evolving practice^(10-12)^. However, there are no studies in the literature that describe the use of a safety checklist in interventional radiology service, an instrument already successfully applied in other countries. In addition, the checklist would help meet Collegiate Board Resolution - CBR 330^(13)^ in its Article 4: “diagnostic or interventional radiology services must implement organizational structure that induces the development of safety culture and continuous improvement of the quality of the structure, processes, and results”.

## OBJECTIVES

To carry out cultural adaptation and validation of the instrument WHO Surgical Safety Checklist: for Radiological Interventions ONLY for Brazilian Portuguese.

## METHODS

### Ethical aspects

The study began after the authors authorized the original version of the instrument, the National Patient Safety Agency, and the approval of the Independent Ethics Committee (IEC). The judges answered the acceptance to participate in the research and sent the free and informed consent form (ICF) signed via e-mail. The signature of the ICF was dispensed to the patients by the IEC since the data collection was only observational in applying the items of the instrument — there was no contact with the patients, and the study did not collect any data from them. The invasive radiological procedure is a daily intervention performed in the hemodynamics unit of the institution, and the study did not alter the execution of the intervention or the routine of the unit.

### Design

A methodological study, guided by the references recommended by the literature^(14-16)^, whose proposals were the cultural adaptation and validation of a patient safety instrument in invasive radiological procedures.

### Original instrument

The original instrument WHO Surgical Safety Checklist: for Radiological Interventions ONLY consists of 28 items divided into three parts, namely: sign in, consisting of 15 items to be completed before the patient is anesthetized (the staff’s understanding about the proposed procedure; questions relating to the patient’s identity and confirmation of his understanding about the procedure to be carried out, as well as his consent to the carry out; conference of the items that are related to the puncture site, and the review of previous imaging examinations; the risks associated with ionizing radiation; a check of the materials and the equipment, checking of the patient’s allergic condition, and the possibility of blood loss, and risk factors for hemorrhage, and renal insufficiency; risk of infection, and venous thromboembolism; staff’s position); time out, consisting of seven items completed before the beginning of the procedure, only in case of general anesthesia (check of the anesthesia apparatus; risk of aspiration; American Society of Anesthesiologists - ASA; monitoring equipment; procedures to avoid infections from the surgical area); and sign out, consisting of six items completed at the end of the procedure before any staff member leaves the room (checking the procedure performed, the instruments and needles used; registration of implanted device; labeling of samples taken; report of problems with the equipment; instructions for post-procedure care for the patient)^(9)^.

**Figure 1:**
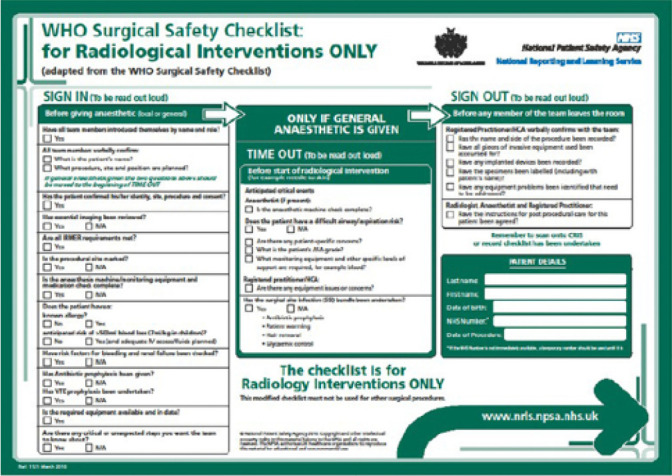
WHO Surgical Safety Checklist: for Radiological Interventions ONLY

*Source: The Royal College of Radiologists. Standards for the NPSA and RCP safety checklist for radiological interventions. [Internet]. 2010. [cited 2020 Jan 02]. Available from: https://www.bsir.org/media/resources/NPSA_RCR_checklist_RCR_2010.pdf*

The original version of the instrument WHO Surgical Safety Checklist: for Radiological Interventions ONLY can be seen in Figure 1.

### Cultural adaptation process

For the cultural adaptation, the study followed six steps: (1) translation of the instrument into the Brazilian Portuguese language; (2) synthesis and achievement of a first consensus of the Portuguese version; (3) evaluation by the committee of judges; (4) back-translation; (5) achievement of a consensus of the English versions and comparison with the original version; and (6) pilot evaluation of the pre-final version (pre-test)^(14-16)^.

In the first stage of the adaptation, two bilingual translators received the instrument to translate it into Portuguese. The translators were Brazilian and laypeople in health care. Then, they synthesized the two initially translated versions, constructing a single version of the two translations. This single version was submitted to the evaluation of a committee of judges for validation of face and content. Five Brazilian judges were selected, fluent in the English language, Doctors with extensive experience in the area of research and knowledge in methodological research.

The parties received the invitation to participate in the instrument validation by email, along with the Informed Consent Form (ICF). After the acceptance and signature of the ICF for experts, the original English version of the instrument was sent by email, as well as the Portuguese version originated from the consensus of the translations for validation. The judges then returned the instrument to the researchers via email with their suggestions.

The research group reached a consensus after a meeting where they performed the validation of face and content after the judges returned all the instruments. Then, judges of the committee received by email the result of this consensus for approval.

The version validated by the judges’ committee was sent for back-translation to two British translators with fluency in Portuguese. The researchers, translators, and back-translators compared the two back-translated versions in terms of wording, grammatical structure, similarity of meaning, and relevance. The discrepancies between the two back-translations and the original instrument were discussed and resolved by consensus among the researchers, resulting in the pre-final Portuguese version of the instrument.

For the pre-test evaluation, the “Portuguese version - Pre-Final” application collected the instrument data in a sample of convenience of ten procedures. At this stage, the instrument evaluated its suitability and applicability. The results of this step were analyzed and submitted to the research group for review. After analysis, it generated the “Portuguese-Final version” of the instrument.

### Analysis of metric properties

One of the ways to assess how reliable an instrument is to analyze its interobserver reliability. It was verified by comparing the checks carried out by two observers^(17)^: two nurses (researcher 1 and researcher 2) by using the “final version” instrument independently and simultaneously in a non-probabilistic sample of 30 procedures. They made the observations after the instrument training and its applicability.

### Period and place of study

The study was developed in the Hemodynamics Unit of a large public teaching hospital, with medium and high complexity care, located in the countryside of the state of Minas Gerais (MG).

The field of study was chosen for the feasibility criterion of carrying out the research since it is a teaching hospital and has a Hemodynamics Unit with two rooms for performing invasive radiological procedures, where cardiac and extracardiac radiological intervention procedures are performed. The hospital did not have implanted patient safety checklist in its routine.

The research collected the pre-test data in September 2019; and the data for the interobserver reliability analysis during October 2019.

### Population or sample

The researchers observed the elective radiological intervention procedures performed in the Hemodynamics Unit of the local institution of the study during the data collection period for the instrument validation. The study sample consisted of ten processes observed in the pre-test and 30 in the interobserver reliability analysis.

### Criteria of inclusion and exclusion

The research included elective radiological intervention procedures such as arteriography or cerebral, iliac, renal, coronary, carotid, and limb angioplasty. It excluded emergency procedures from the study.

### Study protocol

In data collection, there was the observation of the radiological intervention procedure and the completion of the instrument. Two nurses (a master and a post-doctoral student) observed the process inside the hemodynamics room. Both received prior training and are members of the Study and Research Group on Evidence-Based Practice and Patient Safety in the Care Process of the Federal University of Triângulo Mineiro.

### Analysis of results and statistics

For the reliability test, the researchers proceeded with item analysis, which included the absolute and relative frequency distribution of each item of the instrument. The reliability analysis considered the proportion of agreement of the evaluators and, when applicable, the kappa coefficient of agreement, whose values range from 0 (insignificant) to 0.99 (almost perfect)^(18)^.

## RESULTS

Regarding the validation of face and content of the instrument, the research group analyzed the observations made by the judges and accepted the modifications when there was at least 80% agreement among them. Table 1 presents the suggested and accepted changes.

**Table 1:** Amendments suggested by the committee of judges for the creation of the “Portuguese version - Consensus 1” of WHO Surgical Safety Checklist: for Radiological Interventions ONLY, Uberaba, Minas Gerais, Brazil, 2019

Original	Consensus Version	Changes
Title	WHO* Surgical Safety Checklist: only for Radiological interventions	*“*Checklist”
Title	(adapted from the WHO* Surgical List of verifications)	“adapted from Checklist”
Statement	Before anesthetic induction (local or general)	“anesthetic” “(local, regional or general)”
Item 2	The patient’s name?	$left$“What is the”
Item 3	The planned surgical procedure, surgical site and position?	“Are the procedure, surgical site and position planned?”
Item 4	Have the patient confirmed his identity, the surgical site, the procedure and the consent?	“Are the identity of the patient, the type and place of the procedure confirmed and is there one?”
Item 6	Have all the IRMER^†^requirements been met?	“Have all the requirements of the Ionizing Radiation Medical Exposure regulations been met?”
Item 8	Has the anesthesia machine/monitoring equipment and medication been checked?	“been accomplished”
Item 10	Expected risk of blood loss > 500 ml (7 ml/kg in children)? Yes (adequate intravenous access/planned fluids)	“Is there a risk” “venous”
Item 13	Was venous thromboembolic prophylaxis administered??	“performed” “for venous thromboembolism”
Item 14	Is the necessary equipment available and within the expiration date?	“material”
Item 15	Is there any critical or unexpected procedure that you want to communicate to the team?	“any critical step” “unexpected”
Statement	Anesthesiologist (if any):	“if present”
Item 19	What is the ASA^‡^classification of the patient?	“ASA^‡^’s”
Item 20	What monitoring equipment and other specific levels of support are needed, for example blood?	“types”
Item 21	Is there any issue or concern related to equipment?	“concern”
Statement	Remember to record that the verification list was carried out in the medical record.	“the Checklist was carried out”
Statement	The verification list it is only for radiological interventions.	“Checklist” “radiological procedures”
Statement	This modified verification list should not be used in other surgical procedures.	“Checklist” “utilized”

* *WHO - World Health Organization; †IRMER - Ionizing Radiation Medical Exposure Regulations; ‡ASA - American Society of Anesthesiologists.*

In a meeting, it was chosen to keep the name of the instrument WHO Surgical Safety Checklist: for Radiological Interventions ONLY and include “Brazilian Portuguese version.” Without further amendments, they obtained the “Portuguese version - Consensus,” which was forwarded for final approval by the committee of judges. All the judges approved the modifications, without further suggestions.

Then, the “Portuguese version - Consensus” was sent for back-translation, and, after obtaining the pre-final version, it was submitted to the pre-test, in a convenience sample of ten procedures to verify whether the items contained in the instrument would apply to the observed context. In a meeting, the research group discussed the observations made during the pre-test.

Item 4 (“Has the patient confirmed his/her identity, site, procedure and consent?”) was in the pre-final version as: “Estão confirmados: a identidade do paciente, o tipo e o local do procedimento e se há consentimento?” However, the research group understood that it would give the impression that this confirmation would be made with the team, and not with the patient, modifying it to: “O paciente confirmou sua identidade, o sítio cirúrgico, o procedimento e o consentimento?”

The verification by the identification and procedure planning team is already contemplated in items 2 (“What is the patient’s name?”) and 3 (“What procedure, site and position are planned?”). Without further modifications, the final version of the instrument was obtained, which was submitted to the interobserver evaluation.

The reproducibility of the adapted instrument was analyzed using interobserver reliability. In this step, two nurses from the previously trained research group observed, simultaneously and independently, thirty procedures and marked “yes” or “no” for the items checked in the room at the time of the procedure.

Analyzing the results, the researchers observed that the values of the kappa coefficient varied within the classification from moderate to almost perfect agreement (0.535 to 0.933; p < 0.001); and, in the items with 100% agreement, they did not calculate the kappa coefficient because of the perfect agreement.

In 19 of the 28 items of the instrument, the agreement was 100%; the others presented agreement higher than 83%, demonstrating that the items of the instrument were understandable and reliable when applied to the observed context. We emphasize that, during the collection, no patient received general anesthesia, which justifies the designation “does not apply” in items number 16 to 22 of the instrument. The proportion of agreement of the checked items is presented descriptively in Table 2.

**Table 2:** Interobserver reliability analysis of WHO Surgical Safety Checklist: for Radiological Interventions ONLY - Brazilian Portuguese version, Uberaba, Minas Gerais, Brazil, 2019

Item	Yes		No		N/A*		Yes		No		N/A*		Proportion of agreement	Kappa	*p* ^†^
	n	%	n	%	n	%	n	%	n	%	N	%			
1	0	0	30	100	0	0	0	0	30	100	0	0	100	-	-
2	27	90	3	10	0	0	28	93.3	2	6.7	0	0	96.667	0.783	**< 0.001**
3	23	76.7	7	23.3	0	0	20	66.7	10	33.3	0	0	83.333	0.595	**< 0.001**
4	6	20	24	80	0	0	5	16.7	25	83.3	0	0	96.667	0.889	**< 0.001**
5	8	26.7	22	73.3	0	0	8	26.7	22	73.3	0	0	100	-	-
6	0	0	30	100	0	0	30	100	0	0	0	0	100	-	-
7	0	0	30	100	0	0	6	20	24	80	0	0	100	-	-
8	0	0	30	100	0	0	0	0	30	100	0	0	100	-	-
9	7	23.3	23	76.7	0	0	7	23.3	23	76.7	0	0	100	-	-
10	7	23.3	23	76.7	0	0	3	10	27	90	0	0	86.667	0.535	**< 0.001**
11	3	10	27	90	0	0	3	10	27	90	0	0	100	-	-
12	0	0	30	100	0	0	0	0	30	100	0	0	100	-	-
13	13	43.3	17	56.7	0	0	13	43.3	17	56.7	0	0	93.333	0.864	**< 0.001**
14	0	0	30	100	0	0	0	0	30	100	0	0	100	-	-
15	23	76.7	7	23.3	0	0	19	63.3	11	36.7	0	0	86.667	0.689	**< 0.001**
16	0	0	0	0	30	100	0	0	0	0	30	100	100	-	-
17	0	0	0	0	30	100	0	0	0	0	30	100	100	-	-
18	0	0	0	0	30	100	0	0	0	0	30	100	100	-	-
19	0	0	0	0	30	100	0	0	0	0	30	100	100	-	-
20	0	0	0	0	30	100	0	0	0	0	30	100	100	-	-
21	0	0	0	0	30	100	0	0	0	0	30	100	100	-	-
22	0	0	0	0	30	100	0	0	0	0	30	100	100	-	-
23	27	90	3	10	0	0	25	83.3	5	16.7	0	0	93.333	0.714	**< 0.001**
24	0	0	30	100	0	0	0	0	30	100	0	0	100	-	-
25	14	46.7	16	53.3	0	0	15	50	15	50	0	0	96.667	0.933	**< 0.001**
26	1	3.3	29	96.7	0	0	1	3.3	29	96.7	0	0	100	-	-
27	3	10	27	90	0	0	3	10	27	90	0	0	100	-	-
28	27	90	3	10	0	0	28	93.3	2	6.7	0	0	96.667	0.783	**< 0.001**

* *N/A - Not applicable; †p - p value (kappa coefficient).*

**Figure 2:**
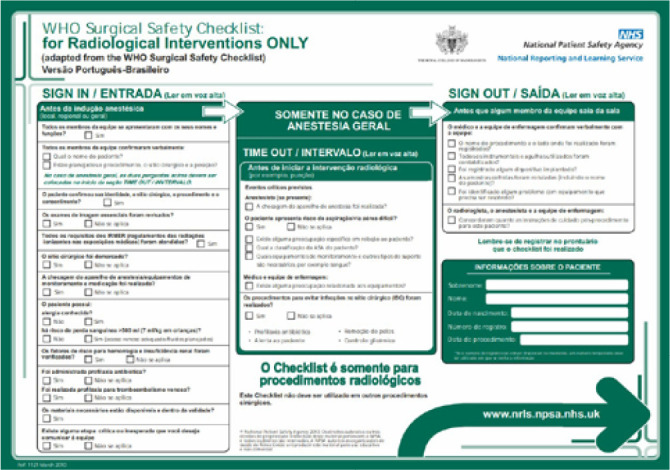
WHO Surgical Safety Checklist: for Radiological Interventions ONLY - Brazilian Portuguese version, Uberaba, Minas Gerais, Brazil, 2019

The Brazilian Portuguese version of the instrument WHO Surgical Safety Checklist: for Radiological Interventions ONLY can be seen in Figure 2.

## DISCUSSION

The cultural adaptation of the instrument WHO Surgical Safety Checklist: for Radiological Interventions ONLY was performed to make it available for its use in Brazil. The study selected the instrument because the WHO recommends the development of new checklists for other in-hospital services as a way to stimulate the safety culture^(5)^ after the initiatives to promote patient safety in surgical procedures, and because there is no checklist specific for interventional radiology services validated for the Brazilian reality.

According to CBR 330, among the management actions of the legal officer of the interventional radiology service, the actions related to safety, the quality of processes, and the protection of patients stand out. Thus, the manager must implement necessary measures to ensure compliance with the requirements of this resolution, such as the development and implementation of tools such as checklists, which promote the early detection of complications and adverse events in the post-intervention period, providing a decrease in complications and better patient safety^(13)^.

The importance of checklists is widely recognized as a crucial step for patient safety. One study reported the experience with the development and implementation of a checklist directed to the radiological intervention activity to limit the probability of errors and harm to patients and assess their impact on the results of the Radiological intervention process. The authors concluded that the introduction of a checklist in the practice of routine radiological intervention was considered feasible and helped to eliminate adverse events during the first year of implementation, generating strong commitment and greater awareness in the health team about patient safety^(7)^.

Evidence shows that the checklist contributes positively to decreases in complications in health care^(19)^. A checklist reduces memory dependency and establishes a mechanism to check for elements that could be forgotten due to human tendencies^(20)^.

In other countries, although still with few publications, the use of a checklist for interventional Radiology has been positive^(21-22)^. It provides staff with communication support, assisting in safe patient care^(23)^. One of the positive points of the tool is the accessibility, ease, and practicality of execution, and can help in opening a communication channel within the multiprofessional team^(24)^.

In 2012 and 2016, the RCR audited the use of the WHO Surgical Safety Checklist for Radiological Interventions ONLY in various modalities and subspecialties of radiological intervention services in the United Kingdom. In 2012, 93% of institutions fully or partially implemented the checklist. In 2016, there was an improvement, with 98% of institutions implementing the tool, and 48% using it for all procedures in all modalities; 50% for some procedures; and 2% did not use it. The process was perceived as effective for patient safety, and the audit pointed out the main limitations for the implementation of the tool: the instrument is not appropriate for minor procedures; lack of staff commitment; and the fact that the checklist is too long and contains some unnecessary data^(25)^.

The authors of a study conducted in Poland analyzed the effect of the checklist in the decrease of adverse events in 2,064 invasive Cardiological and electrophysiological procedures. The use of a checklist was associated with a significant reduction of adverse events, especially bleeding, a decrease in the number of errors related to health care, and positive contribution in the organization and communication within the team^(22)^.

Rafiei et al. described potential items to compose a checklist aimed at invasive radiological procedures and emphasized that the implementation of such a tool requires careful design, effective implementation, teamwork, and management involvement. They also stressed that the pre-procedure checklist is not a panacea, but it is designed to promote communication and encourage team working in a mutual effort to ensure patient safety^(21)^.

However, other studies do not present statistical significance in the reduction of adverse events using a checklist^(24,26)^. The results indicated the lack of teamwork, the business mentality with a focus on speed, and the presence of many items in the checklists as limiting factors for the use of the checklist. As a solution, they suggested a responsible coordinator, the involvement of the entire team, and the possibility of team members requesting a break if they verify the need.

Barriers can contribute to the poor effectiveness of the instrument, such as lack of knowledge about the checklist and its accomplishment; lack of leadership (no member of the team is responsible for promoting and auditing the checklist); staff considers time-consuming and additional bureaucracy; after-hours procedures involving employees from other sectors not familiar with the tool; and loss of instruments. To that end, the nurse can be the principal professional in awareness, training, engagement, and auditing for the implementation of the tool^(27)^.

Dysfunctional communication during care procedures harms team performance, care quality, and patient safety^(28)^. Procedure rooms are historically hierarchical, and this is reflected in the behavior of team members, making it challenging to develop a safety culture^(29)^. Thus, a checklist has the potential to optimize communication, work, and cooperation among the team members; break down hierarchical barriers that are counterproductive to the quality of care and anticipate potential problems^(20,30)^.

In safety checklist models in interventional radiology services proposed in the literature, there is a frequent concern to verify team presentation, history, informed consent, review of previous images, sedation and analgesia, renal function, anticoagulation status, allergies, prior heparin therapy, concerns about equipment, post-procedure instructions and notes performed^(20,22-24,27)^.

It is not the purpose of the checklists to replace the protocols of good clinical practice or to cover all the possibilities of errors in the service but to provide a pause for reflection and discussion before performing any invasive procedure^(6)^.

In interventional radiology, more extensive and multicenter studies are necessary to verify the effectiveness of the use of a checklist and its correlation with the decrease in complications and mortality^(21,27,30)^.

### Study limitations

The instrument WHO Surgical Safety Checklist: for Radiological Interventions Only was not subjected to the process of cultural adaptation in other countries and languages, which made it difficult to discuss the results found in the present study.

Despite this limitation, the instrument is suitable to be adopted in Brazilian services and can contribute to increasing the quality and safety of invasive radiological procedures.

### Contributions to the field of nursing

The adapted version of the instrument WHO Surgical Safety Checklist: for Radiological Interventions ONLY is a tool that health professionals and nurses can apply in the Brazilian context. It provides improvements in clinical practice and team communication, promoting safety for patients undergoing invasive radiological procedures.

## CONCLUSIONS

The process of cultural adaptation and the validation of the instrument WHO Surgical Safety Checklist: for Radiological Interventions ONLY resulted in WHO Surgical Safety Checklist: for Radiological Interventions ONLY - Brazilian Portuguese version.

The translation, cultural adaptation, and validation of face and content of WHO Surgical Safety Checklist: for Radiological Interventions ONLY met the equivalence criteria between the original and the translated instrument. The instrument was understandable and feasible and to be applied by health professionals in invasive radiological procedures in Brazil.

## Supplementary Material

0034-7167-reben-75-06-e20210011-sup01

## SUPPLEMENTARY MATERIAL

Article extracted from the master’s dissertation “Cultural adaptation and validation of the instrument WHO Surgical Safety Checklist: for Radiological Interventions ONLY; version for Brazilian Portuguese”, presented to Federal University of Triângulo Mineiro, Uberaba, MG, Brazil.

The dissertation is available in the repository of the Digital Library of theses and dissertations of the Federal University of Triângulo Mineiro and can be accessed by the following link: http://bdtd.uftm.edu.br/handle/tede/1032.
