# Supplementary material for: Validation of a patient safety checklist for radiological procedures in hemodynamics
Source: Rev Bras Enferm. 2022 May 11;75(6):e20210011. doi: 10.1590/0034-7167-2021-0011 (PMC9728881; doi:10.1590/0034-7167-2021-0011)
Supplement: 0034-7167-reben-75-06-e20210011-sup01 [file 0034-7167-reben-75-06-e20210011-sup01.pdf]

Universidade Federal do Triângulo Mineiro

Programa de Pós-Graduação *Stricto Sensu* em Atenção à Saúde

Flávia Ana Pacheco

Adaptação cultural e validação do instrumento *WHO Surgical Safety Checklist: for Radiological Interventions ONLY* – versão para o português brasileiro

Uberaba/MG

2020

Universidade Federal do Triângulo Mineiro

Programa de Pós-Graduação *Stricto Sensu* em Atenção à Saúde

Flávia Ana Pacheco

Adaptação cultural e validação do instrumento *WHO Surgical Safety Checklist: for Radiological Interventions ONLY* – versão para o português brasileiro

Dissertação apresentada ao Programa de Pós-Graduação *Stricto Sensu* em Atenção à Saúde da Universidade Federal do Triângulo Mineiro, como parte dos requisitos para a obtenção do título de Mestre em Atenção à Saúde.

Orientadora: Profa. Dra. Maria Helena Barbosa

Linha de Pesquisa: Atenção à Saúde das Populações

Eixo Temático: Saúde do Adulto e do Idoso

Uberaba/MG

2020

Catalogação na fonte:
Biblioteca da Universidade Federal do Triângulo Mineiro

Pacheco, Flávia Ana
P119a Adaptação cultural e validação do instrumento *WHO Surgical Safety Checklist: for Radiological Interventions ONLY* – versão para o português brasileiro / Flávia Ana Pacheco. -- 2020.
95 f.: il., tab.

Dissertação (Mestrado em Atenção à Saúde) -- Universidade Federal do Triângulo Mineiro, Uberaba, MG, 2020
Orientadora: Profa. Dra. Maria Helena Barbosa

1. Segurança do paciente. 2. Radiologia intervencionista. 3. Estudos de validação. 4. Pacientes - Cuidado e tratamento. I. Barbosa, Maria Helena. II. Universidade Federal do Triângulo Mineiro. III. Título.

CDU 616-083

Cicera Daniele da Silva - CRB6/3012

Autorizo a reprodução e divulgação total ou parcial deste trabalho, por qualquer meio convencional ou eletrônico, para fins de estudo e pesquisa, desde que citada a fonte.

Flávia Ana Pacheco

Adaptação cultural e validação do instrumento *WHO Surgical Safety Checklist: for Radiological Interventions ONLY* – versão para o português brasileiro

Dissertação apresentada ao Programa de Pós-Graduação *Stricto Sensu* em Atenção à Saúde da Universidade Federal do Triângulo Mineiro, como parte dos requisitos para a obtenção do título de Mestre em Atenção à Saúde.

______ de ________________ de ________

Banca Examinadora:

____

Profa. Dra. Maria Helena Barbosa – Orientadora

Universidade Federal do Triângulo Mineiro

____

Profa. Dra. Elizabeth Barichello

Universidade Federal do Triângulo Mineiro

____

Prof. Dra. Patrícia da Silva Pires

Universidade Federal da Bahia

Aos meus amores Sofia e Rone. Em memória dos pacientes que, por falhas em sua assistência, perderam suas vidas. Que estas mortes não tenham sido em vão. Que possamos aprender com os erros e evitar eventos futuros**.**

"Foi o tempo que dedicastes à tua rosa que a fez tão importante."

Antonie de Saint-Exupéry

AGRADECIMENTOS

A Deus, essencial em minha vida, por iluminar meus caminhos.

À professora Dra. Maria Helena Barbosa, por dividir sua vasta sabedoria, pela paciência, orientação e pelo incentivo, que tornaram possível o desenvolvimento da pesquisa, e, sobretudo, me fez uma pessoa certamente melhor, como seu exemplo.

A todos os professores da Pós-Graduação em Atenção a Saúde, em especial ao Dr. Vanderlei José Haas, por seu incansável incentivo, apoio e orientação.

Às pós-doutorandas Márcia Marques Felix e Maria Beatriz Guimarães, que enchem meu coração de orgulho. Vocês me ajudaram, incentivaram e orientaram. Levarei vocês eternamente em meu coração

Aos secretários do Programa de Pós-Graduação *Stricto Sensu* em Atenção à Saúde Daniele Cristina Marques Machado e Fábio Renato Barboza, por todo auxílio e incentivo. Vocês são muito especiais e tornaram cada dia desta jornada mais leve.

A todos os integrantes do Grupo de Pesquisa da professora Dra. Maria Helena Barbosa. Cada um de vocês contribuiu para meu desenvolvimento. É muito bom ser uma de vocês.

Aos meus 22 amigos de mestrado. Aprendi muito com cada um de vocês e os levarei em meu coração, em especial à minha dupla, Isadora Braga Calegari. Minha querida, obrigada por tudo.

Aos funcionários da Hemodinâmica do Hospital das Clínicas da Universidade Federal do Triângulo Mineiro, que me acolheram, ajudaram e incentivaram em todas as partes da pesquisa, em especial ao Dr. Fernando de Martino e à Enfermeira Gabriela Lucas Cardoso.

Aos pacientes que, direta ou indiretamente, participaram desta pesquisa.

Ao meu pai e à minha mãe.

RESUMO

Discussões sobre segurança do paciente nas unidades hospitalares configuram uma tendência mundial. Nos Estados Unidos, estima-se que, anualmente, ocorram 251 mil mortes por complicações advindas de erros assistenciais, o que representa 9,5% das mortes no país, sendo a terceira maior causa de mortalidade, atrás apenas das doenças cardiovasculares e do câncer. No Brasil, apesar de ainda subnotificados, óbitos por erros assistenciais são uma realidade e representam 0,6% do total dos eventos adversos notificados. Em 2009, a *World Health Organization* publicou recomendações para promover a segurança do paciente em procedimentos cirúrgicos. Sua campanha "Cirurgia Segura Salva Vidas" introduziu o conceito de um *checklist*, o *Surgical Safety Checklist*, destinado a identificar e controlar os riscos durante as três fases do procedimento cirúrgico: antes da indução da anestesia, antes da incisão da pele e antes da saída da sala de operações. A utilização desse *checklist* mostrou-se significativa na redução da morbimortalidade. A radiologia intervencionista é uma especialidade com incidência de complicações e de morbimortalidade menor em comparação aos procedimentos cirúrgicos, por sua natureza minimamente invasiva. Entretanto, esses procedimentos têm muitos aspectos em comum com procedimentos cirúrgicos (complexidade, rápida resolução, urgência e emergência, trabalho em equipe etc.) e, consequentemente, implicam em risco potencial para falhas e complicações. Implementar uma lista de verificações em serviços de radiologia intervencionista pode ter a mesma eficácia em segurança do paciente que os *checklists* cirúrgicos. Este estudo teve como objetivo realizar a adaptação cultural e a validação do instrumento *WHO Surgical Safety Checklist: for Radiological Interventions ONLY* para o português brasileiro. Trata-se de uma pesquisa metodológica cujo processo de adaptação cultural percorreu as seguintes etapas: tradução do instrumento para o idioma português brasileiro, síntese e obtenção do primeiro consenso da versão em português, avaliação pelo comitê de juízes, retrotradução, obtenção do consenso das versões em inglês e comparação com a versão original e pré-teste. O instrumento *WHO Surgical Safety Checklist: for Radiological Interventions ONLY* é constituído por 28 itens divididos em três partes componentes, sendo elas: *sign in*, composto por 15 itens a serem preenchidos antes de o paciente ser anestesiado (seja anestesia geral ou regional); *time out*, composto por sete itens, preenchidos antes do início do procedimento, apenas em caso de

anestesia geral; e *sign out*, composto por seis itens, preenchidos ao término do procedimento, antes que qualquer membro da equipe saia da sala. A análise de confiabilidade considerou a proporção de concordância dos avaliadores e o coeficiente de concordância Kappa. Os resultados evidenciaram que os valores do coeficiente Kappa variaram de moderado a quase perfeito (0,535 a 0,933; p>0,001), e em 19, dos 28 itens do instrumento, a concordância foi de 100% − os demais apresentaram concordância maior que 83%, demonstrando que os itens do instrumento foram compreensíveis e confiáveis quando aplicados ao contexto observado. A tradução, a adaptação cultural e a validação de face e conteúdo do *WHO Surgical Safety Checklist: for Radiological Interventions ONLY* satisfez os critérios de equivalência entre o instrumento original e o traduzido. O instrumento mostrou-se compreensível e viável, podendo ser aplicado em procedimentos radiológicos invasivos no Brasil. Ressalta-se a importância de realizar estudos futuros, que avaliem a aplicação do instrumento para verificar a necessidade de readequação da versão aqui disponibilizada.

Palavras-Chaves: Segurança do Paciente. Radiologia Intervencionista. Estudos de Validação.

ABSTRACT

Discussions about patient safety in hospital units are a worldwide trend. In the United States only it is estimated that 251,000 deaths from complications due to care errors occur annually, representing 9.5% of deaths in the country, and being the third leading cause of mortality behind cardiovascular disease and cancer. In Brazil, although still underreported, deaths from care errors are a reality and represent 0.6% of the total adverse events reported. In 2009, World Health Organization issued recommendations to promote patient safety undergoing surgical procedures. Its “Safe Surgery Saves Lives” campaign introduced the concept of a checklist, the Surgical Safety Checklist, designed to identify and control risks during the three stages of the surgical procedure: before anesthetic induction, before skin incision and before leaving the operating room. The use of this checklist proved to be significant in reducing morbimortality. Interventional radiology is a specialty with lower incidence of complications and morbimortality, compared to surgical procedures, due to its minimally invasive nature. However, these procedures have many aspects in common with surgical procedures (complexity, rapid resolution, urgency and emergency, teamwork etc.) and, consequently, imply a potential risk for failures and complications. Implementing a checklist in interventional radiology can be as effective in patient safety as surgical checklists. This study aimed to perform the cultural adaptation and validation of the WHO Surgical Safety Checklist: for Radiological Interventions ONLY instrument for Brazilian Portuguese. It is about a methodological research whose process of cultural adaptation went through the following steps: translation of the instrument into the Brazilian Portuguese language, synthesis - obtaining the first consensus of the Portuguese version, evaluation by the judges committee, feedback translation, consensus acquisition of the versions in English and comparison with the original and pretest version. WHO Surgical Safety Checklist: for Radiological Interventions ONLY instrument consists of 28 items divided into three component parts, namely: sign in, consisting of 15 items to be completed before the patient is anesthetized (either general or regional anesthesia); time out, consisting of seven items that will be completed before the procedure begins, only for general anesthesia and sign out, consisting of six items that will be completed at the end of the procedure before any staff member leaves the room. The reliability analysis considered the raters’ agreement

ratio and the Kappa agreement coefficient. The results highlighted that the Kappa coefficient values ranged from moderate to almost perfect (0.535 to 0.933; $p>0.001$) and in 19 of the 28 items of the instrument the agreement was 100% − the others showed agreement higher than 83%, showing that the instrument items were understandable and reliable when applied to the observed context. The translation, cultural adaptation, and face and content validation of the WHO Surgical Safety Checklist: for Radiological Interventions ONLY met the equivalence criteria between the original instrument and the translated one. The instrument proved to be understandable and viable and could be applied in invasive radiological procedures in Brazil. The importance of conducting future studies that evaluate the application of the instrument to verify the need for readjustment of the version available here is highly recommended.

Keywords: Patient Safety. Radiology, Interventional. Validation Studies.

RESUMEN

Discusiones acerca de la seguridad del paciente en las unidades hospitalarias se constituyen como una tendencia mundial. En Estados Unidos, se estima que, anualmente, ocurran 251 mil muertes por complicaciones oriundas de errores asistenciales, lo que representa un 9,5% de las muertes en el país, siendo la tercera mayor causa de mortalidad, traspasada solamente por las enfermedades cardiovasculares y el cáncer. Ya en Brasil, aunque no totalmente notificadas, las muertes por errores asistenciales se tratan de una realidad y representan un 0,6% del total de los eventos adversos informados. En 2009, la *World Health Organization* publicó recomendaciones para promover la seguridad del paciente en procedimientos quirúrgicos. Su campaña "Cirugía Segura Salva Vidas" introdujo el concepto de un *checklist*, el *Surgical Safety Checklist*, con la función de identificar y controlar los riesgos a lo largo de las tres fases del procedimiento quirúrgico: antes de la inducción de la anestesia, antes de la incisión de la piel y antes de la salida del quirófano. La utilización de ese *checklist* fue significativa en la reducción de la morbimortalidad. La radiología intervencionista se trata de una especialidad con baja incidencia de complicaciones y de morbimortalidad cuando comparada a los procedimientos quirúrgicos, debido a su naturaleza mínimamente invasiva. Sin embargo, dichos procedimientos tienen muchos aspectos semejantes a los procedimientos quirúrgicos (complejidad, rápida resolución, urgencia y emergencia, trabajo en equipe, etc.) y, consecuentemente, conllevan un potencial riesgo para fallos y complicaciones. Implementar una lista de verificaciones en servicios de radiología intervencionista puede presentar la misma eficacia hacia la seguridad del paciente que los *checklists* quirúrgicos. Este estudio tuvo como objetivo realizar la adaptación cultural y la validación del instrumento *WHO Surgical Safety Checklist: for Radiological Interventions ONLY* para el portugués brasileño. Dicha investigación metodológica cuyo proceso de adaptación cultural recorrió las siguientes etapas: traducción del instrumento para el idioma portugués brasileño, síntesis y obtención del primero consenso de la versión en portugués, evaluación por el comité de jueces, traducción inversa, obtención del consenso de las versiones en inglés y comparación con la versión original y pre-test. El instrumento *WHO Surgical Safety Checklist: for Radiological Interventions ONLY* es constituido por 28 ítems divididos en tres partes componentes, siendo ellas: *sign in*, compuesto por quince ítems que deben ser

rellenados antes que sea anestesiado el paciente (sea anestesia general o regional); *time out*, compuesto por siete ítems, rellenados antes del comienzo del procedimiento, solamente en caso de anestesia general, y *sign out*, compuesto por seis ítems, rellenados al término del procedimiento, antes que salga de la sala cualquier miembro del equipo. El análisis de fiabilidad consideró la proporción de concordancia de los evaluadores y el coeficiente de concordancia Kappa. Los resultados señalaron que los valores del coeficiente Kappa oscilaban entre moderados y casi perfectos (0,535 a 0,933; p>0,001), y en 19, de los 28 ítems del instrumento la concordancia fue de un 100% - los demás presentaron concordancia mayor que un 83%, demostrando que los ítems del instrumento fueron comprensibles y fiables cuando aplicados al contexto observado. La traducción, la adaptación cultural y la validación de la faz y contenido del *WHO Surgical Safety Checklist: for Radiological Interventions ONLY* satisfizo los criterios de equivalencia entre el instrumento original y el traducido. El instrumento se mostró comprensible y viable, así que puede ser aplicado en procedimientos radiológicos invasivos en Brasil. Se hace hincapié a la importancia de la realización de estudios futuros que evalúen la aplicación del instrumento para verificar la necesidad de readecuación de la versión disponible aquí.

Palabras-clave: Seguridad del Paciente. Radiología Intervencional. Estudios de Validación.

LISTA DE ILUSTRAÇÕES

**Figura 1**	Processo de adaptação cultural do instrumento *WHO Surgical Safety Checklist: for Radiological Interventions ONLY*. Uberaba, MG, Brasil, 2020.	34

LISTA DE QUADROS

**Quadro 1**	Itens do *WHO Surgical Safety Checklist: for Radiological Interventions ONLY*. Uberaba, MG, Brasil, 2020.	24
**Quadro 2**	Interpretação dos valores Kappa. Uberaba, MG, Brasil, 2020.	40
**Quadro 3**	Caracterização dos membros integrantes do grupo de juízes. Uberaba, MG, Brasil, 2020.	43
**Quadro 4**	Alterações sugeridas pelo comitê de juízes para criação da Versão Português Consenso 1 do instrumento *WHO Surgical Safety Checklist: for Radiological Interventions ONLY,* Uberaba, MG, Brasil, 2020.	44

LISTA DE TABELAS

Tabela 1 Análise da confiabilidade interobservadores do instrumento *WHO Surgical Safety Checklist: for Radiological Interventions ONLY*. Uberaba, MG, Brasil, 2020 48

LISTA DE SIGLAS

CEP	Comitê de Ética em Pesquisa
CIRSE	*Cardiovascular and Interventional Radiological Society of Europe*
CNPq	Conselho Nacional de Desenvolvimento Científico e Tecnológico
NPSA	*National Patient Safety Agency*
RADPASS	*Radiological Patient Safety System*
RCR	*Royal College of Radiologists*
SPSS	*Statistical Package for the Social Science*
SSC	*Surgical Safety Checklist*
TCLE	Termo de Consentimento Livre e Esclarecido
UFTM	Universidade Federal do Triângulo Mineiro
VF	Versão Final
VI	Versão Inglês
VO	Versão Original
VP	Versão Português
VPC	Versão Português Consenso
VPF	Versão Português Pré-Final
WHO	*World Health Organization*

SUMÁRIO

**1**	**INTRODUÇÃO**	20
1.1	*WHO SURGICAL SAFETY CHECKLIST: FOR RADIOLOGICAL INTERVENTIONS ONLY*	23
**2**	**JUSTIFICATIVA**	30
**3**	**HIPÓTESE**	31
**4**	**OBJETIVOS**	32
4.1	*OBJETIVO GERAL*	32
4.2	*OBJETIVOS ESPECÍFICOS*	32
**5**	**MATERIAL E MÉTODOS**	33
5.1	*DELINEAMENTO DO ESTUDO*	33
5.2	*PERMISSÃO PARA ADAPTAÇÃO CULTURAL, VALIDAÇÃO E USO DO INSTRUMENTO DE CHECKLIST*	33
5.3	*PROCESSO DE ADAPTAÇÃO CULTURAL*	33
**5.3.1**	**Etapa 1: tradução**	35
**5.3.2**	**Etapa 2: síntese e obtenção do primeiro consenso da versão em português**	35
**5.3.3**	**Etapa 3: validade de face e conteúdo por comitê de juízes**	35
**5.3.4**	**Etapa 4: retrotradução**	36
	**Etapa 5: obtenção do consenso das versões em inglês e comparação com a versão original**	36
**5.3.5**	**Etapa 6: avaliação da Versão Pré-Final**	36
5.4	*ETAPA RELACIONADAS ÀS PROPRIEDADES PSICOMÉTRICAS*	37

**5.4.1**	**Análise da confiabilidade interobservadores**	37
**5.4.2**	**Campo de estudo**	37
**5.4.3**	**População e amostra**	38
**5.4.4**	**Critérios de inclusão e exclusão**	38
**5.4.5**	**Coleta de dados**	39
5.4.5.1	*Procedimentos de coleta pré-teste*	39
5.4.5.2	*Procedimentos de coleta interobservadores*	39
**5.4.6**	**Análise de dados**	40
**5.4.7**	**Aspectos éticos**	41
**6**	**RESULTADOS**	42
6.1	*PROCESSO DE ADAPTAÇÃO CULTURAL*	42
**6.1.1**	**Tradução**	42
**6.1.2**	**Consenso da versão em português**	42
**6.1.3**	**Comitê de juízes**	42
**6.1.4**	**Retrotradução**	46
**6.1.5**	**Obtenção do consenso das versões em inglês e comparação com a versão original**	46
**6.1.6**	**Pré-teste**	46
6.2	*CONFIABILIDADE INTEROBSERVADORES*	47
**7**	**DISCUSSÃO**	50
**8**	**CONCLUSÃO**	55
	REFERÊNCIAS	56
**ANEXO A**	*WHO Surgical Safety Checklist: for Radiological Interventions ONLY*	65
**ANEXO B**	Autorização da *National Patient Safety Agency* para utilização do instrumento	66
**ANEXO C**	Autorização do Comitê de Ética em Pesquisa	67
**ANEXO D**	Versão atualizada do *WHO Surgical Safety Checklist: for Radiological Interventions ONLY*	73
**APÊNDICE A**	Termo de Consentimento Livre e Esclarecido (TCLE) − comitê de juízes	74
**APÊNDICE B**	Carta-convite para composição do comitê de juízes	77

**APÊNDICE C**	Versões Português 1 e 2	78
**APÊNDICE D**	Versão Português Consenso 1	85
**APÊNDICE E**	Versão Português Consenso 2	86
**APÊNDICE F**	Versões Inglês 1 e 2	87
**APÊNDICE G**	Versão Pré-Final	94
**APÊNDICE H**	Versão Final	95

1 INTRODUÇÃO

As discussões sobre segurança do paciente nas unidades hospitalares configuram tendência mundial (DUARTE *et al*., 2015). Nos Estados Unidos, estima-se que, anualmente, ocorram 251 mil mortes por complicações advindas de erros assistenciais, o que representa 9,5% das mortes no país, constituindo a terceira maior causa de mortalidade, atrás apenas das doenças cardiovasculares e do câncer (ANDERSONG e ABRAHAMSON, 2017; MAKARY e DANIEL, 2016). No Brasil, apesar de ainda subnotificados, óbitos por erros assistenciais são uma realidade e representam 0,6% do total dos eventos adversos notificados (MAIA *et al*., 2018).

Em 2009, a *World Health Organization* (WHO) publicou iniciativas para promover a segurança do paciente em procedimentos cirúrgicos. Sua campanha Cirurgia Segura Salva Vidas introduziu o conceito de um *checklist*, *o Surgical Safety Checklist* (SSC), destinado a identificar e controlar os riscos durante as três fases do procedimento cirúrgico: antes da indução da anestesia, antes da incisão da pele e antes da saída da sala de operações (WHO, 2009).

O SSC é uma inovação simples, que tem sido bem recebida pela equipe cirúrgica. Sua utilização mostrou-se significativa na redução da morbimortalidade em estudo que avaliou a utilização desse *checklist* em hospitais com diversidades econômicas, de localização e de sistemas de cuidados de saúde (HAYNES *et al*., 2009). Os autores compararam dados de 3.733 pacientes antes da introdução do *checklist* com dados de 3.955 após sua introdução, resultando na queda da taxa de mortalidade de 1,5% para 0,8% e da taxa de complicação de 11 para 7%.

Estudos subsequentes também evidenciaram reduções da mortalidade e da morbidade com a utilização do SSC (HAUGEN *et al*., 2015; VAN KLEI *et al*., 2012). No entanto, nem todos os estudos suportam tais conclusões, como evidenciado na pesquisa de Urbach (2014), desenvolvida em Ontário, a qual não relatou mudanças em termos de resultados pós-operatórios com a implementação do SSC.

Esses achados divergentes sugerem que a maneira como os hospitais implementam o SSC é fundamental para sua efetividade. No estudo de Urbach *et al*. (2014), 92 dos 101 hospitais estudados não modificaram o instrumento para atender suas necessidades (LEAPE, 2014). Para o sucesso da implementação do *checklist*, é necessário que a equipe seja treinada e sensibilizada quanto a sua utilização. Para

melhorar a segurança do paciente, a implementação da lista de verificação exige cooperação entre anestesistas, cirurgiões e enfermeiros (KORKIAKANGAS, 2017). A WHO recomenda a adaptação do *checklist* de acordo com a realidade de cada instituição, abordagem essa que promove um sentido de apropriação do instrumento e melhor adesão ao *checklist* (WHO, 2009).

A radiologia intervencionista é uma especialidade com incidência de complicações e de morbimortalidade menor em comparação aos procedimentos cirúrgicos, por sua natureza minimamente invasiva (WONG *et al*., 2015). Entretanto, procedimentos radiológicos invasivos têm muitos aspectos em comum com procedimentos cirúrgicos (complexidade, rápida resolução, urgência e emergência, trabalho em equipe etc.) e, consequentemente, implicam em risco potencial para falhas e complicações. Assim, implementar uma lista de verificações em radiologia intervencionista pode ter a mesma eficácia em segurança do paciente que os *checklists* cirúrgicos (CORSO *et al*., 2014).

Barbosa *et al*. (2013), em estudo prospectivo realizado com 127 pacientes submetidos a procedimentos radiológicos invasivos, observaram que 79 (62,2%) pacientes apresentaram complicações. Destes, 39 (49,4%) apresentaram complicações locais, 25 (31,7%) sistêmicas e 15 (18,9%) tiveram complicações locais e sistêmicas associadas.

Dentre os eventos que têm maior incidência em serviços de radiologia intervencionista estão aqueles relacionados à identificação do paciente, à suspensão dos exames devido à falta de preparo do paciente, a deficiências no atendimento ao paciente, a informações deficientes ou a conclusão ilegível de documentos (BOSCÁ-MAYANS *et al*., 2012).

Ainda, a unidade de radiologia intervencionista frequentemente recebe o paciente direcionado de outro setor. Dessa forma, geralmente o contato da equipe da radiologia intervencionista com o paciente antes do procedimento é curto, o que torna difícil para o médico ou o enfermeiro reunir todas as informações clínicas necessárias. Entretanto, questões específicas, como a função renal e a determinação da história alérgica, são essenciais, bem como as informações ao paciente sobre possíveis reações tardias. Nesse sentido, a implantação de uma lista de verificação de segurança em radiologia intervencionista poderia evitar muitas complicações e problemas potenciais (WONG *et al*., 2015). Os mesmos autores ressaltam que, como

um benefício secundário, o *checklist* em radiologia intervencionista também pode servir como importante ferramenta de comunicação entre os membros da equipe.

Em 2009, a *National Patient Safety Agency* (NPSA) e o *Royal College of Radiologists* (RCR) adaptaram o *checklist* Cirúrgica Segura da WHO para um *checklist* específico para intervenções radiológicas para a Inglaterra e o País de Gales, intitulado *WHO Surgical Safety Checklist: for Radiological Interventions ONLY* (THE ROYAL COLLEGE OF RADIOLOGISTS, 2009) (Anexo A). Tais órgãos fizeram observações sobre o uso do *checklist*, a saber: trata-se de um método simples e de custo eficaz para melhorar a segurança do paciente; o tempo gasto no preenchimento é de menos de 1 minuto; um julgamento inicial com um pequeno grupo motivado dentro do setor é recomendado, pois permite modificações para atender a prática local; a modificação e a otimização da lista de verificação para procedimentos específicos aumenta a aceitabilidade da lista de verificação para os radiologistas; a inclusão de radiologistas e enfermeiros é essencial para garantir que as listas de verificação sejam concluídas; a digitalização do formulário é eficiente para futuras conclusões sobre sua implantação; e o uso da lista de verificação deixa o paciente mais seguro quanto ao procedimento *(*THE ROYAL COLLEGE OF RADIOLOGISTS, 2013*).*

A adesão ao *checklist* como parte de uma cultura de segurança pela equipe é essencial. A NPSA e o RCR aconselham e encorajam a adaptação do *checklist* para atender às necessidades locais *(*THE ROYAL COLLEGE OF RADIOLOGISTS, 2010*).*

Neste sentido, grupos têm trabalhado em adaptações do *WHO Surgical Safety Checklist: for radiological Interventions ONLY*. Em 2010, um grupo na Holanda criou o *RADiological Patient Safety System* (RADPASS), baseado no instrumento da WHO e na opinião de especialistas. Com a implementação do *checklist*, a média de situações não verificadas corretamente caiu de 24% na pré-implementação para 5% na pós-implementação. Também não ocorreram mais adiamentos e cancelamentos, que somavam em 10% anteriormente (KOETSER *et al*., 2013).

Com base no *checklist* da WHO, no RADPASS e em uma equipe de especialistas internacionais, a *Cardiovascular and Interventional Radiological Society of Europe* (CIRSE) produziu uma lista de verificação para radiologia intervencionista, a qual é fortemente recomenda. Essa lista de verificação é ajustável para atender às necessidades dos diferentes centros e foi desenvolvida para ser aplicável em uma variedade de procedimentos radiológicos invasivos. A lista de verificação consiste em

32 itens, divididos em três seções (planejamento, entrada e saída), com três respostas possíveis (‘sim’, ‘não’ e ‘não aplicável’) (LEE *et al*., 2012). Corso *et al*. (2014) mostraram que o uso de um *checklist* baseado no instrumento da CIRSE foi viável e eficaz, colaborando com a diminuição de eventos adversos e promovendo a participação e a conscientização de segurança do paciente, entre a equipe responsável por procedimentos radiológicos invasivos.

1.1 *WHO SURGICAL SAFETY CHECKLIST: FOR RADIOLOGICAL INTERVENTIONS ONLY*

O WHO Surgical Safety Checklist: for Radiological Interventions ONLY é constituído por 28 itens divididos em três partes componentes, sendo elas: *sign in*, composta por 15 itens a serem preenchidos antes de o paciente ser anestesiados (seja anestesia geral ou regional); *time out*, composta por sete itens preenchidos antes do início do procedimento, apenas em caso de anestesia geral; *e sign out*, composta por seis itens preenchidos ao término do procedimento antes que qualquer membro da equipe saia da sala.

No Quadro 1, apresentam-se os itens do instrumento *WHO Surgical Safety Checklist: for Radiological Interventions ONLY*.

Quadro 1 − Itens do *WHO Surgical Safety Checklist: for Radiological Interventions ONLY*. Uberaba, MG, Brasil, 2020.

SIGN IN<br><br>***Before giving anaesthetic*** *(local or general)*	
Item 1	*Have all team members introduced themselves by name and role?*
Item 2	*What is the patient’s name?*
Item 3	*What procedure, site and position are planned?*
Item 4	*Has the patient confirmed his/her identity, site, procedure and consent?*
Item 5	*Has essential imaging been reviewed?*

Item 6	*Are all IRMER requirements met?*
Item 7	*Is the procedural site marked?*
Item 8	*Is the anaesthesia machine/monitoring equipment and medication check complete?*
Item 9	*Does the patient have a: known allergy?*
Item 10	*Anticipated risk of >500mL blood loss (7mL/kg in children)? Yes (and adequate IV access/fluids planned)*
Item 11	*Have risk factors for bleeding and renal failure been checked?*
Item 12	*Has antibiotic prophylaxis been given?*
Item 13	*Has VTE prophylaxis been undertaken?*
Item 14	*Is the required equipment available and in date?*
Item 15	*Are there any critical or unexpected steps you want the team to know about?*
TIME OUT<br>***Before start of radiological intervention*** *(for example: needle to skin)*	
Item 16	*Is the anaesthetic machine check complete?*
Item 17	*Does the patient have a difficult airway/aspiration risk?*
Item 18	*Are there any patient-specific concerns?*
Item 19	*What is the patient’s ASA grade?*
Item 20	*What monitoring equipment and other specific levels of support are required, for example blood?*
Item 21	*Are there any equipment issues or concerns?*
Item 22	*Has the SSI bundle been undertaken?*

	• *Antibiotic prophylaxis*<br>• *Patient warming*<br>• *Hair removal*<br>• *Glycaemic control*
SIGN OUT<br>***Before any member of the team leaves the room***	
Item 23	*Has the name and side of the procedure been recorded?*
Item 24	*Have all pieces of invasive equipment used been accounted for?*
Item 25	*Have any implanted devices been recorded?*
Item 26	*Have the specimens been labelled (including with patient's name)?*
Item 27	*Have any equipment problems been identified that need to be addressed?*
Item 28	*Have the instructions for post procedural care for this patient been agreed?*

Fonte: Guidelines for radiologists in implementing the NPSA Safe Surgery requirement. The Royal College of Radiologists, London, fev. 2009. Disponível em: https://www.bsir.org/media/resources/NPSA_checklist_guidance_RCR_2009.pdf. Acesso em: 2 jan. 2020.
Nota: IRMER: *Ionising Radiation (Medical Exposure) Regulations*; VTE: *venous thromboembolism*; ASA: *American Society of Anesthesiologists*; *SSI: surgical site infection*.

O item 1 ("*Have all team members introduced themselves by name and role?*") enfatiza o entendimento da equipe sobre o procedimento proposto. Nos itens 2 *(*"*What is the patient's name?*") e 4, ("*Has the patient confirmed their identity, the procedure, site and consent?*") são abordadas questões referentes à identificação do paciente e à confirmação do conhecimento do paciente acerca do procedimento a ser realizado, bem como seu consentimento para realização.

Observam-se frequentemente fatores agravantes para equívocos relacionados à identificação do paciente, como incidência de homônimos e parônimos. Erros neste sentido são realidade e têm potencial para serem a causa raiz de inúmeros desfechos catastróficos, tanto ao paciente quanto ao profissional e ao estabelecimento de saúde. Destaca-se, assim, a necessidade da conferência e da pronúncia correta do nome completo (TASE *et al*., 2018).

Os itens 3 (“*What procedure, site and position are planned?*”), 4 (“*Has the patient confirmed his/her identity, site, procedure and consent?*”) e 7 (“*Is the procedural site marked?*”) abordam a conferência de itens relacionados ao adequado local de punção para a melhor realização do procedimento. Se a demarcação do local não for usual no serviço, a equipe precisa discutir e acordar sobre o local escolhido para punção e o local a ser examinado ou tratado (THE ROYAL COLLEGE OG RADIOLOGISTS, 2013).

Em cirurgia segura, a demarcação correta e clara da pele, no pré-operatório, mostra-se como medida simples e eficaz para evitar erros relacionados ao local do procedimento (BATHLA *et al*., 2018). Na radiologia intervencionista, a demarcação do sítio a ser puncionado e do local-alvo a ser examinado ou tratado é pouco usual, visto que, para a maioria dos procedimentos, a demarcação do local a ser examinado ou tratado é pouco efetiva. Por exemplo, em um planejamento para angioplastia da artéria renal direita, teoricamente a pele sobre o rim direito deveria ser demarcada. No entanto, após o preparo do paciente, o local é coberto por campo estéril; desta forma, a marca não seria visível. Nestes casos, recomenda-se que o lado da intervenção seja confirmado da maneira tradicionalmente usada por radiologistas: por imagem no intraprocedimento (ANGLE *et al*., 2008).

O consentimento informado é essencial para a assistência centrada no paciente. Ele enfatiza a importância de o paciente participar ativamente de seus cuidados. Toda exposição médica à radiação ionizante deve ser justificada e autorizada antes de ser considerada. É um desafio para a equipe, com o pequeno contato com o paciente, realizar as explicações, para que ele compreenda os riscos potenciais dos agentes de radiação e contraste, sangramento ou infecção (RIPLEY *et al*., 2015).

O item 4 (“*Has the patient confirmed his/her identity, site, procedure and consent?*”) valoriza a confirmação do próprio paciente de seus dados e consentimento. O envolvimento do paciente durante a conferência de seus dados pode reduzir o medo e aumentar seu empoderamento. É importante criar condições em que os pacientes sejam incentivados a fazerem perguntas ou colocações durante o *checklist*; não falar sobre o paciente como se ele não estivesse presente, mas manter contato com suas preocupações (BERGS *et al*., 2018).

O item 5 (“*Has essential imaging been reviewed?*”) aborda a necessidade de revisão de exames de imagem prévios, o que pode ser muito útil para orientar o

procedimento. Esta revisão é capaz de diminuir o tempo de procedimento e o uso de agente de contraste, e impedir complicações. Em alguns serviços, é possível a exibição de estudos prévios nos monitores, para revisão contínua durante um procedimento (RAFIEI *et al*., 2016).

Procedimentos radiológicos invasivos expõem pacientes e trabalhadores de saúde à radiação ionizante, que, em doses excessivas ou acumulativas, pode causar danos celulares definitivos e prejudiciais (MOURA e BACCHIM NETO, 2015). O item 6, ("*Are all IRMER [Ionising Radiation (Medical Exposure) Regulations] requirements met*?"), é destinado à verificação de itens para proteção da equipe e dos pacientes aos riscos associados à radiação ionizante, sendo eles: dosimetria e proteção adequada da equipe, que inclui o uso de avental de chumbo, protetor de tireoide e de olhos; nível de radiação ao qual o paciente será exposto; avaliação de possíveis riscos e potenciais benefício em crianças; e investigação de mulheres em idade fértil, para verificar a possibilidade de gestação. Este item é claramente irrelevante para procedimentos que não envolvem radiação ionizante e pode ser omitido em listas de verificação de procedimentos guiados por ultrassom (MOURA e BACCHIM NETO, 2015; THE ROYAL COLLEGE OF RADIOLOGISTS, 2013; BRITISH INSTITUTE OF RADIOLOGY *et al*., 2015). O questionamento quanto à gestação pode ser um assunto delicado, principalmente a menores de 16 anos. O serviço deve considerar o melhor momento e local para tal questionamento, garantindo a privacidade das pacientes (BRITISH INSTITUTE OF RADIOLOGY *et al*, 2015).

Os materiais e equipamentos devem ser conhecidos e verificados regularmente (THE ROYAL COLLEGE OF RADIOLOGISTS, 2013). A conferência dessa verificação é realizada pela checagem dos itens 8 ("*Is the anaesthesia machine/monitoring equipment and medication check complete*?"), 14 ("*Is the required equipment available and in date?*"), 16 ("*Is the anaesthetic machine check complete?*"), 20 ("*What monitoring equipment and other specific levels of support are required, for example blood?*"), 24 ("*Have all pieces of invasive equipment used been accounted for?*") e 27 ("*Have any equipment problems been identified that need to be addressed?*").

Durante o procedimento radiológico invasivo é frequente a utilização de opioides, ansiolíticos, contrastantes e antibióticos profilático, o que resulta em um potencial risco de alergia. Verificar alergias antes do início do procedimento pode evitar complicações por reações alérgicas (CORNELIS *et al*., 2019; RAFIEI *et al*.,

2016). O item 9 tem o propósito de checar a situação alérgica do paciente (“*Does the patient have a: known allergy*?”).

O item 10 (“*anticipated risk of >500mL blood loss (7mL/kg in children)? Yes (and adequate IV access/fluids planned)*”) verifica a possibilidade de perda sanguínea. O sangramento após o procedimento radiologico invasivo é comum e está associado com significativa morbimortalidade, devendo este ser considerado antes, durante e após o procedimento, com foco em estratégias para reduzir o risco para o paciente (GEORGE *et al*., 2016).

O uso de contraste intravascular pode induzir à nefropatia aguda. Fatores como diabetes, doença renal crônica, insuficiência cardíaca congestiva e/ou idade avançada elevam potencialmente o risco para a nefropatia induzida pelo contraste (CHAWLA *et al*., 2017). O item 11 (“*Have risk factors for bleeding and renal failure been checked?*”) checa fatores de risco para hemorragia e insuficiência renal.

Nos itens 12 (“*Has antibiotic prophylaxis been given?*”) e 22 (“*Has the SSI bundle been undertaken? Antibiotic prophylaxis, Patient warming, Hair removal, Glycaemic control*”), são verificados itens referentes ao risco de infecção. Apesar da ocorrência relativamente rara de complicações infecciosas e de poucas evidências sobre o uso de antibioticoprofilaxia na radiologia intervencionista, esta pode ser positiva em alguns casos (CHEHAB e*t al*., 2018). Os *bundles* são ações conjuntas de boas práticas. Os estudos ainda são controversos quanto ao uso de *bundles* no desfecho para prevenção de infecção em procedimentos (ANDERSON *et al*., 2014; ANDRADE *e*t *al*., 2019).

Prever o risco de tromboembolismo venoso é desafiador. Há vários fatores a serem considerados, como câncer, obesidade, reposição hormonal, imobilização prolongada e trombofilias (PATEL *et al*., 2019). A checagem da profilaxia de tromboembolismo é verificada no item 13 (“*Has VTE prophylaxis been undertaken?”*).

No item 15 (“*Are there any critical or unexpected steps you want the team to know about*?”) há um incentivo adicional para que qualquer pessoa da equipe de radiologia intervencionista exponha uma questão de segurança. Esse item tem potencial para evitar erros e melhorar atitudes de segurança e trabalho em equipe. Ao existir esta abertura para que a equipe faça suas colocações, evita-se que, por apreensão ou embaraços, alguma informação importante deixe de ser exposta (RAFIEI *et al*., 2016).

Os itens 16 ao 22 devem ser preenchidos apenas se o paciente for submetido à anestesia geral, que é pouco frequente em radiologia intervencionista. Geralmente, sedação e anestesia local são consideradas como ideais, pela preservação da cooperação e do conforto do paciente, e pela duração reduzida do procedimento, além de evitar o comprometimento respiratório − que contribui para piores resultados clínicos e custos mais altos. O desfecho desejado para sedação e analgesia deve ser discutido e planejado antes do procedimento (CORNELIS *et al*., 2019; THE AMERICAN SOCIETY OF ANESTHESIOLOGISTS, 2018; URMAN *et al*., 2019)

Os itens 23 (“*Has the name and side of the procedure been recorded?*”), 24 (“*Have any implanted devices been recorded?*”) e o 26 (“*Have the specimens been labelled (including with patient’s name)?*”) referem-se às corretas identificações do procedimento ocorrido, dos dispositivos implantados e do material coletado. Aguiar *et al*. (2016), em estudo que objetivou verificar as complicações ocorridas e os principais cuidados de enfermagem no cateterismo cardíaco, observaram subregistro dos cuidados realizados durante o procedimento.

O último item do instrumento, o 28 (“*Have the instructions for post procedural care for this patient been agreed?*”) refere-se à concordância das instruções de cuidado pós-procedimento.

2 JUSTIFICATIVA

No Brasil, o *checklist* em cirurgias é uma prática em evolução (ALPENDRE *et al*., 2017; DIEGO *et al*., 2016; MAZIERO, 2015). No entanto, ainda não há publicação na literatura brasileira sobre a implantação de uma lista de verificações de segurança em serviço de radiologia intervencionista, instrumento este já aplicado com êxito em outros países.

O cuidado para não causar dano ao paciente é um dever dos profissionais de saúde. O *checklist* é uma ferramenta que pode auxiliar na segurança, na prática clínica e na comunicação da equipe (RAFIEI *et al*., 2016).

Frente ao exposto, é importante a condução do presente estudo com foco na validação do instrumento *WHO Surgical Safety Checklist: for Radiological Interventions ONLY*, que pode ser aplicado no contexto brasileiro, tendo como benefício a melhoria da segurança do paciente submetido a procedimentos radiológicos invasivos.

3 HIPÓTESE

A lista de verificações WHO *Surgical Safety Checklist: for Radiological Interventions ONLY* é adaptável ao contexto brasileiro, podendo constituir ferramenta útil para segurança do paciente submetido a procedimentos radiológicos invasivos.

4 OBJETIVOS

4.1 OBJETIVO GERAL

- Realizar a adaptação cultural e a validação do instrumento *WHO Surgical Safety Checklist: for Radiological Interventions ONLY* para o português brasileiro.

4.2 OBJETIVOS ESPECÍFICOS

- Realizar a tradução e a retrotradução do instrumento *WHO Surgical Safety Checklist: for Radiological Interventions ONLY*.
- Avaliar a validade de face e conteúdo da versão para o português brasileiro do *WHO Surgical Safety Checklist: for Radiological Interventions ONLY* por meio da análise de um comitê de juízes.
- Avaliar a confiabilidade interobservadores da versão adaptada do instrumento *WHO Surgical Safety Checklist: for Radiological Interventions ONLY* em sua versão para o português brasileiro.

5 MATERIAL E MÉTODOS

5.1 DELINEAMENTO DO ESTUDO

Trata-se de pesquisa metodológica, cujas propostas foram a adaptação cultural e a validação de um instrumento de segurança do paciente em procedimentos radiológicos invasivos. Este tipo de pesquisa refere-se aos estudos sobre métodos de obtenção, organização e análise dos dados, e trata da elaboração, validação e avaliação de instrumentos e técnicas de investigação (POLIT e BECK, 2018).

5.2 PERMISSÃO PARA ADAPTAÇÃO CULTURAL, VALIDAÇÃO E USO DO INSTRUMENTO DE CHECKLIST

Inicialmente, solicitou-se a autorização à NPSA para realização desta pesquisa. Em resposta a tal solicitação, um e-mail foi recebido no dia 24 de agosto de 2016, com a autorização para que o instrumento fosse adaptado para o idioma português do Brasil (Anexo B).

5.3 PROCESSO DE ADAPTAÇÃO CULTURAL

Para a adaptação cultural, foi utilizado os referenciais de Guillemin *et al*. (1993), Ferrer *et al.* (1996) e Dantas (2007), abrangendo as seguintes etapas: (1) tradução do instrumento *WHO Surgical Safety Checklist: for Radiological Interventions ONLY* para o idioma português brasileiro; (2) síntese e obtenção do primeiro consenso da versão em português; (3) avaliação pelo comitê de juízes; (4) retrotradução; (5) obtenção do consenso das versões em inglês e comparação com a versão original (VO); e (6) avaliação piloto da Versão Pré-Final (pré-teste). A Figura 1 apresenta um fluxograma das etapas do processo de adaptação transcultural.

Figura 1 – Processo de adaptação cultural do instrumento *WHO Surgical Safety Checklist: for Radiological Interventions ONLY*. Uberaba, MG, Brasil, 2020.

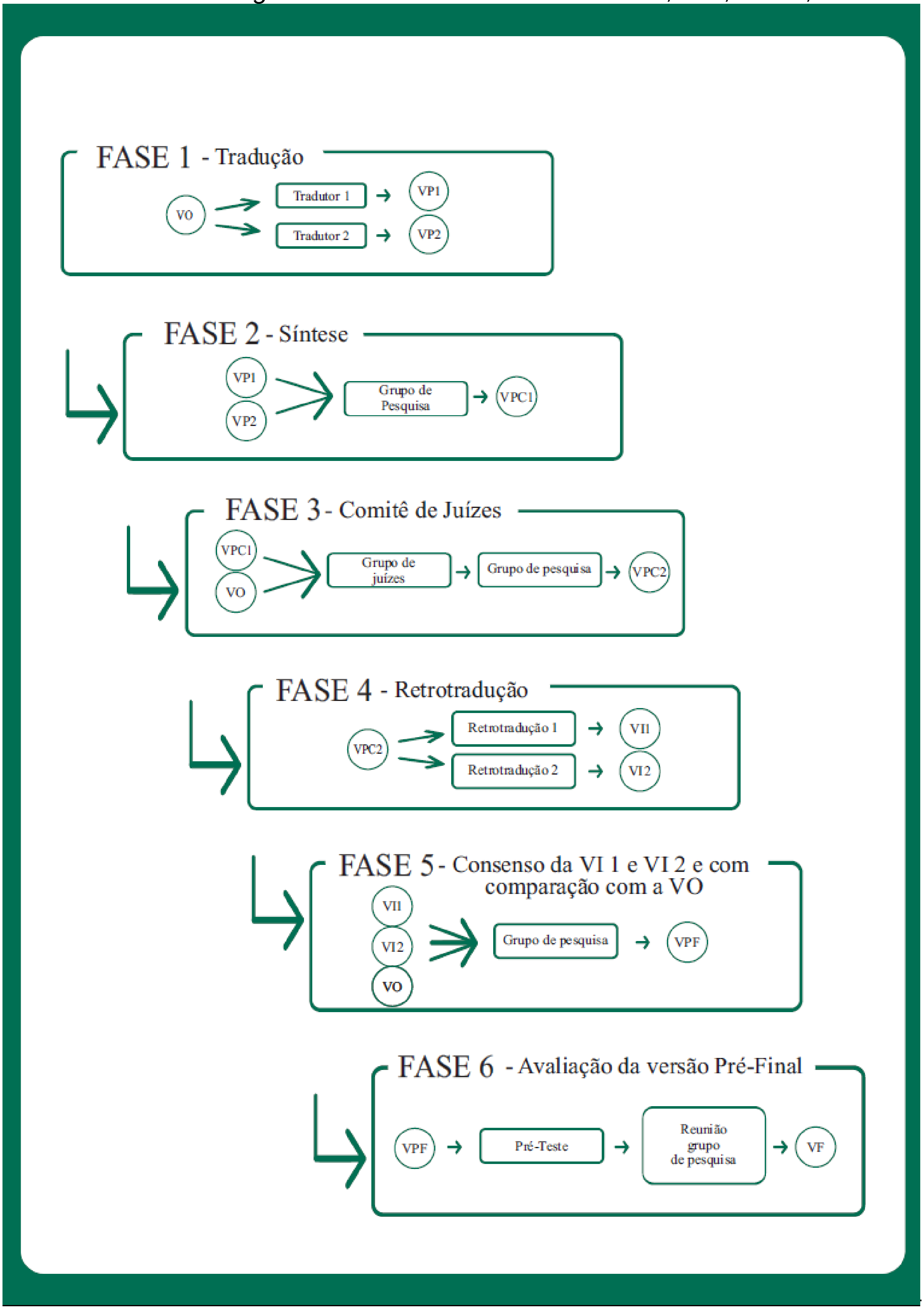

Fonte: Autora, 2020.
Nota: VO: Versão Original; VP: Versão Português; VPC: Versão Português Consenso; VI: Versão Inglês; VPF: Versão Pré-Final; VF: Versão Final.

5.3.1 Etapa 1: tradução

A tradução do instrumento *WHO Surgical Safety Checklist: for Radiological Intervencions ONLY* em sua VO para língua portuguesa foi realizada por dois tradutores de língua inglesa, brasileiros e leigos em assistência à saúde. Cada um elaborou uma versão em português, as quais denominamos de Versão Português 1 (VP1) e Versão Português 2 (VP2).

5.3.2 Etapa 2: síntese e obtenção do primeiro consenso da versão em português

A síntese das traduções VP1 e VP2 foi realizada por um consenso entre o grupo de pesquisa e os tradutores, originando a Versão Português Consenso 1 (VPC1).

5.3.3 Etapa 3: validade de face e conteúdo por comitê de juízes

A validação refere-se ao grau que um instrumento mede aquilo que supõe estar medindo. Existem três tipos de validade de um instrumento: de conteúdo, de constructo e relacionada a um critério (POLIT e BECK, 2018). Nesse estudo, foi realizada a validade de conteúdo, que analisa o conteúdo do instrumento, ou seja, verifica se os itens constituem representativamente aquilo que se propõem avaliar.

Para compor o comitê de juízes, foram selecionados cinco juízes brasileiros, fluentes em língua inglesa, sendo eles dois enfermeiros, dois médicos e um pesquisador com compreensão em pesquisa metodológica.

Primeiramente, os juízes foram convidados via *e-mail*, por convite que continha o Termo de Consentimento Livre e Esclarecido (TCLE) (Apêndice A) com a descrição da finalidade e dos objetivos da pesquisa e a carta-convite (Apêndice B). Os juízes responderam o aceite em participar da pesquisa e encaminharam o TCLE assinado também via *e-mail*. Após recebido o consentimento dos juízes, os pesquisadores encaminharam um segundo *e-mail* a eles com a VO e a VPC1, para avaliação do instrumento, no que se refere à validade de face e conteúdo. Após, os juízes devolveram o instrumento aos pesquisadores via *e-mail*, com suas sugestões.

A validade de conteúdo foi verificada pelo consenso obtido em reunião com o grupo de pesquisa, após a devolução de todos os instrumentos pelos juízes, na sede do Programa de Pós-Graduação *Stricto Sensu* em Atenção à Saúde. Em seguida, este consenso foi encaminhado via *e-mail* para aprovação dos juízes do comitê. Após a aprovação dos juízes, originou-se a VPC2.

5.3.4 Etapa 4: retrotradução

A VPC2 foi encaminhada, via *e-mail*, para retrotradução, para dois tradutores de origem britânica e com fluência na língua portuguesa. Esses profissionais realizaram as retrotraduções de forma individual, obtendo-se a Versão Inglês 1 (VI1) e a Versão Inglês 2 (VI2).

5.3.5 Etapa 5: obtenção do consenso das versões em inglês e comparação com a versão original

As duas versões retrotraduzidas (VI1 e VI2) foram comparadas pelos pesquisadores, tradutores e os retrotradutores, com relação à redação, à estrutura gramatical, à similaridade de significado e à relevância. As discrepâncias entre as duas traduções e/ou as retrotraduções e o instrumento original foram discutidas e resolvidas por consenso entre os pesquisadores, para obter a Versão Português Pré-Final (VPF).

5.3.6 Etapa 6: avaliação da Versão Pré-Final

Para avaliação pré-teste, a coleta de dados foi realizada por meio da aplicação da versão adaptada do instrumento, a VPF, em uma amostra de conveniência de dez procedimentos. Nesta etapa, o instrumento é aplicado, para que se possam avaliar sua adequabilidade e aplicabilidade. Os resultados dessa etapa foram resumidos e submetidos ao grupo de pesquisa para revisão. Após análise, gerou-se a Versão Final (VF).

5.4 ETAPA RELACIONADAS ÀS PROPRIEDADES PSICOMÉTRICAS

5.4.1 Análise da confiabilidade interobservadores

Uma das formas de se avaliar o quanto um instrumento é confiável consiste em analisar sua confiabilidade interobservadores. Para tanto, dois ou mais observadores treinados são envolvidos, fazendo observações simultâneas e independentes. Quando os escores dados por dois observadores independentes a um mesmo fenômeno mostram-se congruentes, provavelmente esses escores são precisos e confiáveis (POLIT e BECK, 2018).

A confiabilidade interobservadores foi verificada por comparação das checagens realizadas por dois observadores, sendo duas enfermeiras (pesquisador 1 e pesquisador 2), ao utilizarem o instrumento VF de maneira independente e simultânea, em uma amostra não probabilística de 30 procedimentos. As observações foram realizadas após treinamento com o instrumento e sua aplicabilidade.

5.4.2 Campo de estudo

O estudo foi desenvolvido em um hospital público de ensino de grande porte, com 302 leitos, com atendimentos de média e alta complexidade, localizado no interior do estado de Minas Gerais, Região Sudeste do Brasil, e referência para o município e região do Triângulo Sul, composta por 27 municípios (HC/UFTM, 2016).

O hospital possuía uma unidade na qual são realizados procedimentos radiológicos invasivos, denominada Unidade de Hemodinâmica, com duas salas de procedimentos. Foram realizados, em média, 159 procedimentos eletivos/mês, segundo dados obtidos junto à secretaria da referida unidade, de janeiro a dezembro de 2017. A equipe era composta por 36 pessoas: 14 técnicos em enfermagem, seis médicos cardiologistas intervencionistas, cinco enfermeiros, quatro cardiologistas especialistas em marca-passo, três técnicos em radiologia, dois médicos vascular, um neurointervencionista e um eletrofisiologista.

A escolha do campo de estudo baseou-se no critério de viabilidade de realização da pesquisa, por tratar-se de um hospital de ensino e apresentar uma Unidade de Hemodinâmica com duas salas para realização de procedimentos radiológicos invasivos. O hospital não possuía lista de verificações de segurança do paciente na referida unidade implantada em sua rotina.

5.4.3 População e amostra

A população-alvo para a validação do instrumento foram os procedimentos realizados em pacientes internados e ambulatoriais submetidos a procedimento eletivo de intervenção radiológica. A assinatura do TCLE foi dispensada pelo Comitê de Ética em Pesquisa (CEP). A amostra do estudo consistiu de cinco juízes, que avaliaram a clareza e a compreensão dos itens do instrumento, dez procedimentos observados no pré-teste e 30 na análise de confiabilidade interobservadores.

5.4.4 Critérios de inclusão e exclusão

Os critérios de inclusão para os juízes foram: ser brasileiro; com domínio na língua inglesa; possuírem título de Doutor; um dos juízes ser graduado em enfermagem, com reconhecido saber em qualidade e segurança do paciente; dois dos juízes serem graduados em medicina, um anestesiologista atuante em unidades de radiologia intervencionista ou hemodinamicista; dois dos juízes serem graduados em área de saúde, com reconhecido saber em estudos metodológicos com validação de instrumentos; estar inserido em atividades de pesquisas na temática; ter interesse expresso em colaborar com o estudo.

Os dados relativos à titulação dos profissionais foram verificados a partir do Currículo *Lattes*.

Os critérios de inclusão para os procedimentos foram: realizados em pacientes com idade igual ou superior a 18 anos de ambos os sexos; de arteriografia ou angioplastia cerebral, de coronárias, de carótidas, de membros, ilíaca e renal, realizados na Unidade de Hemodinâmica da instituição sede do estudo durante o período da coleta de dados.

Foram excluídos do estudo procedimentos realizados em caráter emergencial.

5.4.5 Coleta de dados

Para a coleta de dados, foi realizada observação do procedimento, e foram feitas anotações quanto à aplicabilidade do instrumento. As observações foram realizadas dentro da sala de hemodinâmica, no momento de realização do procedimento. Os dados do pré-teste foram coletados no mês de setembro de 2019, e os dados para a análise de confiabilidade interobservadores foram coletados durante o mês de outubro de 2019.

5.4.5.1 Procedimentos de coleta pré-teste

Uma enfermeira do grupo de pesquisa observou a realização de dez procedimentos e verificou se os itens contidos na VPF se aplicariam ao contexto observado.

5.4.5.2 Procedimentos de coleta interobservadores

Duas enfermeiras do grupo de pesquisa (pesquisador 1 e pesquisador 2) utilizaram o instrumento VF de maneira independente e simultânea, na amostra de 30 procedimentos. As observações foram realizadas após treinamento quanto ao instrumento e à sua aplicabilidade.

5.4.6 Análise de dados

Um banco de dados foi construído, as variáveis foram codificadas em um dicionário (*codebook*), e os dados foram digitados em planilhas eletrônicas, adotando-se a técnica de dupla digitação, com posterior validação. A planilha validada foi importada, empregando-se o aplicativo *Statistical Package for the Social Science* (SPSS), versão 23.0, utilizado também na análise exploratória e inferencial.

Para o alcance do primeiro objetivo específico, foi adotado o processo de adaptação cultural, segundo referencial adotado.

O segundo objetivo específico foi alcançado pela avaliação do comitê de juízes, considerando validos os itens com pelo menos 80% de concordância entre os juízes.

Para o terceiro objetivo específico de testar a confiabilidade, foi realizada a análise de itens, que incluiu a distribuição de frequência absoluta e relativa de cada item do instrumento. A análise de confiabilidade considerou a proporção de concordância dos avaliadores e, quando aplicável, o coeficiente de concordância Kappa. Para expressar a força de concordância do coeficiente Kappa e a interpretação dos valores Kappa, foi utilizada a classificação exposta no Quadro 2.

Quadro 2 − Interpretação dos valores Kappa. Uberaba, MG, Brasil, 2020.

**Coeficiente KAPPA**	**Concordância**
0	Insignificante
0,01-0,20	Leve/ baixa
0,21-0,40	Regular
0,41-0,60	Moderada
0,61-0,80	Substancial
0,81-0,99	Quase perfeita

Fonte: LANDIS, J. R.; KOCH, G. G. The measurement of observer agrément for categorical data. **Biometrics**, v.33, p. 159-174, 1977.

5.4.7 Aspectos éticos

Em cumprimento da Resolução 466/2012 do Conselho Nacional de Saúde para pesquisas envolvendo seres humanos, o estudo foi aprovado pelo CEP da Universidade Federal do Triângulo Mineiro (UFTM), sob o número 3.109.638/2019 (Anexo C).

O TCLE para os juízes foi obtido via *e-mail* pelo pesquisador responsável, após aprovação deste projeto nas devidas instâncias éticas.

Houve dispensa da obtenção do TCLE junto aos pacientes pelo CEP, visto que foi realizado apenas observação do procedimento radiológico invasivo e anotações quanto à aplicabilidade do instrumento, não foi foram obtidas informações do paciente e a realização do procedimento é uma rotina no setor (Anexo C).

6 RESULTADOS

6.1 PROCESSO DE ADAPTAÇÃO CULTURAL

6.1.1 Tradução

Cada um dos dois tradutores de língua inglesa geraram uma versão traduzida para o português do *WHO Surgical Safety Checklist: for Radiological Interventions ONLY*, a VP1 e a VP2 (Apêndice C).

6.1.2 Consenso da versão em português

O grupo de pesquisa, em reunião, analisou a VP1 e a VP2 e, em consenso, selecionou as frases mais assertivas, obtendo-se a VPC1 (Apêndice D).

6.1.3 Comitê de juízes

Um grupo de cinco juízes foi constituído por integrantes selecionados por meio da Plataforma Lattes do Conselho Nacional de Desenvolvimento Científico e Tecnológico (CNPq), entre Doutores, enfermeiros e médicos, com *expertise* ou publicações relacionadas em pelo menos uma das áreas: radiologia intervencionista, segurança do paciente e/ou validação de instrumento. A caracterização do grupo de juízes está descrita no Quadro 3.

Quadro 3 − Caracterização dos membros integrantes do grupo de juízes. Uberaba, MG, Brasil, 2020.

Juiz	Caracterização
**1**	Graduação e doutorado em medicina, residência em clínica médica, cardiologia e hemodinâmica. Médico hemodinamicista e professor adjunto de cardiologia
**2**	Graduação, mestrado e doutorado em enfermagem, professora associada, experiência em validação de instrumentos
**3**	Graduação, mestrado e doutorado em enfermagem, especialização em administração hospitalar e em centro cirúrgico, professora associada. experiência em validação de instrumentos e em segurança do paciente
**4**	Graduação em medicina, mestrado e doutorado em anestesiologia, residência em anestesiologia. Médica anestesiologista com atuação em hemodinâmica e professora associada
**5**	Graduação, mestrado e doutorado em enfermagem. Experiência hemodinâmica

Fonte: Elaborado pela autora, 2020.

Quanto às características sociodemográficas dos peritos, três (66,66%) eram do sexo feminino, com tempo de formação entre 27 e 38 anos. No que concerne às áreas de atuação profissional, dois (40%) estavam envolvidos com assistência, ensino e pesquisa; dois (40%) com ensino e pesquisa; e um (25%) com assistência e pesquisa.

A VPC1 e a VO foram encaminhadas via *e-mail* a cada membro do comitê de juízes, os quais realizaram a avaliação das equivalências cultural, semântica, conceitual e idiomática entre os documentos enviados. Suas observações foram encaminhadas via *e-mail* para a pesquisadora. As alterações sugeridas e acatadas estão apresentadas no Quadro 4.

Quadro 4 − Alterações sugeridas pelo comitê de juízes para criação da Versão Português Consenso 1 do instrumento *WHO Surgical Safety Checklist: for Radiological Interventions ONLY,* Uberaba, MG, Brasil, 2020.

Original	Versão consenso	Alterações
**Título**	Lista de verificação de segurança cirúrgica da OMS: somente para intervenções radiológicas	"*Checklist*"
**Título**	(adaptada da lista de verificações cirúrgicas da OMS)	"adaptado do *Checklist*"
**Enunciado**	Antes da indução anestésica (local ou geral)	"anestésica (local, regional ou geral)"
**Item 2**	O nome do paciente?	"Qual é o"
**Item 3**	O procedimento cirúrgico, o sítio cirúrgico e a posição planejados?	"Estão planejados o procedimento, o sítio cirúrgico e a posição?"
**Item 4**	O paciente confirmou sua identidade, o sítio cirúrgico, o procedimento e o consentimento?	"Estão confirmados: a identidade do paciente, o tipo e o local do procedimento e se há?"
**Item 6**	Todos os requisitos dos IRMER (regulamentos das radiações ionizantes nas exposições médicas) foram atendidos?	"Todos os requisitos dos regulamentos das radiações ionizantes nas exposições médicas foram atendidos?"
**Item 8**	A checagem do aparelho de anestesia/equipamentos de monitoramento e medicação foi feita?	"foi realizada"
**Item 10**	Risco previsto de perda sanguínea >500mL (7mL/kg em crianças)? Sim (acesso endovenoso adequado/fluidos planejados)	"Há risco" "venoso"

**Item 13**	Foi administrada profilaxia tromboembólica venosa?	“realizada” “para tromboembolismo venoso”
**Item 14**	Os equipamentos necessários estão disponíveis e dentro da validade?	“materiais”
**Item 15**	Existe algum procedimento crítico ou inesperado que você deseja comunicar à equipe?	“alguma etapa crítica” “inesperada”
**Enunciado**	Anestesista (se houver):	“se presente”
**Item 19**	Qual a classificação ASA do paciente?	“da ASA”
**Item 20**	Quais equipamentos de monitoramento e outros níveis específicos de suporte são necessários, por exemplo sangue?	“tipos”
**Item 21**	Existe alguma questão ou preocupação relacionada aos equipamentos?	“preocupação”
**Enunciado**	Lembre-se de registrar no prontuário que a lista de verificações foi realizada	“o *Checklist* foi realizado”
**Enunciado**	A lista de verificações é somente para intervenções radiológicas	“*Checklist*” “procedimentos radiológicos”
**Enunciado**	Esta lista de verificações modificada não deve ser usada em outros procedimentos cirúrgicos	“*Checklist*” “utilizado”

Fonte: Elaborado pela autora, 2020.
Nota: Trechos grifados indicam que sofreram alterações.

OMS: Organização Mundial da Saúde; IRMER: *Ionising Radiation (Medical Exposure) Regulations*; ASA: *American Society of Anesthesiologists*.

As observações realizadas pelos juízes foram analisadas pelo grupo de pesquisa, e as modificações foram acatadas pelo pesquisador, quando houve pelo menos 80% de concordância entre os juízes. Em reunião, optou-se por manter o nome do instrumento *WHO Surgical Safety Checklist: for Radiological Interventions Only* e

incluir ‘Versão Português-Brasileiro’. Sem mais alterações, obteve-se a VPC2 (Apêndice E), que foi reencaminhada para aprovação final do comitê de juízes. Todos os juízes aprovaram as modificações, sem outras sugestões.

6.1.4 Retrotradução

A VPC2 foi encaminhada via *e-mail* para retrotradução, para dois tradutores de origem britânica e com fluência na língua portuguesa e foram obtidas a VI1 e a VI2.

6.1.5 Obtenção do consenso das versões em inglês e comparação com a versão original

As duas versões retrotraduzidas (VI1 e VI2) (Apêndice F) foram comparadas pelos pesquisadores, tradutores e retrotradutores com relação à redação, à estrutura gramatical, à similaridade de significado e à relevância. As discrepâncias entre as duas retrotraduções e o instrumento original foram discutidas e resolvidas por consenso entre os pesquisadores, obtendo-se a VPF (Apêndice G).

6.1.6 Pré-teste

A VPF foi submetida ao pré-teste em uma amostra de conveniência de dez procedimentos. Em reunião, o grupo de pesquisa discutiu as observações realizadas durante o pré-teste.

O item 4 (“*Has the patient confirmed his/her identity, site, procedure and consent?*”) estava na VPF como: “Estão confirmados: a identidade do paciente, o tipo e o local do procedimento e se há consentimento?”, e o grupo de pesquisa entendeu que daria a impressão de que essa confirmação seria feita com a equipe, e não com o paciente, o modificando para: “O paciente confirmou sua identidade, o sítio cirúrgico, o procedimento e o consentimento?”.

A checagem pela equipe da identificação e planejamento do procedimento já é contemplada nos itens 2 (“*What is the patient’s name*?”) e 3 (“*What procedure, site and position are planned*?”).

Sem outras modificações, obteve-se, então, a VF (Apêndice H), que foi submetida à avaliação interobservadores.

6.2. CONFIABILIDADE INTEROBSERVADORES

A reprodutibilidade do instrumento adaptado foi analisada por meio da confiabilidade interobservadores. Nesta etapa, dois enfermeiros do grupo de pesquisa previamente treinados observaram, de forma simultânea e independente, 30 procedimentos e assinalaram ‘sim’ ou ‘não’ para os itens verificados em sala no momento do procedimento.

Os valores do coeficiente Kappa variaram de moderado a quase perfeito (0,535 a 0,933; $p>0,001$) e, nos itens em que houve 100% de concordância, o coeficiente Kappa não foi calculado, pois ocorreu a concordância perfeita.

A proporção de concordância dos itens checados está apresentada descritivamente na Tabela 1. Em 19 dos 28 itens do instrumento, a concordância foi de 100%; os demais apresentaram concordância maior que 83%, demonstrando que os itens do instrumento eram compreensíveis e confiáveis, quando aplicados ao contexto observado. Ressaltamos que, durante a coleta, nenhum paciente foi submetido à anestesia geral, o que justifica o “não se aplica” nos itens de número 16 a 22 do instrumento.

Tabela 1 - Análise da confiabilidade interobservadores do instrumento *WHO Surgical Safety Checklist: for Radiological Interventions ONLY*. Uberaba, MG, Brasil, 2020

**Itens**	**Observador 1**			**Observador 2**			**Proporção de concordância**	**Kappa**	**Valor de p**
	**Sim**	**Não**	**NA**	**Sim**	**Não**	**NA**			
1	0	30 (100)	0	0	30 (100)	0	100	-	-
2	27 (90,0)	3 (10,0)	0	28 (93,3)	2 (6,7)	0	96,667	0,783	>0,001
3	23 (76,7)	7 (23,3)	0	20 (66,7)	10 (33,3)	0	83,333	0,595	>0,001
4	6 (20)	24 (80,0)	0	5 (16,7)	25 (83,3)	0	96,667	0,889	>0,001
5	8 (26,7)	22 (73,3)	0	8 (26,7)	22 (73,3)	0	100	-	-
6	0	30 (100)	0	30 (100)	0	0	100	-	-
7	0	30 (100)	0	6 (20,0)	24 (80,0)	0	100	-	-
8	0	30 (100)	0	0	30 (100)	0	100	-	-
9	7 (23,3)	23 (76,7)	0	7 (23,3)	23 (76,7)	0	100	-	-
10	7 (23,3)	23 (76,7)	0	3 (10,0)	27 (90,0)	0	86,667	0,535	>0,001
11	3 (10,0)	27 (90,0)	0	3 (10,0)	27 (90,0)	0	100	-	-
12	0	30 (100)	0	0	30 (100)	0	100	-	-
13	13 (43,3)	17 (56,7)	0	13 (43,3)	17 (56,7)	0	93,333	0,864	>0,001
14	0	30 (100)	0	0	30 (100)	0	100	-	-
15	23 (76,7)	7 (23,3)	0	19 (63,3)	11 (36,7)	0	86,667	0,689	>0,001
16	0	0	30 (100)	0	0	30 (100)	100	-	-
17	0	0	30 (100	0	0	30 (100)	100	-	-

18	0	0	30 (100)	0	0	30 (100)	100	-	-
19	0	0	30 (100)	0	0	30 (100)	100	-	-
20	0	0	30 (100)	0	0	30 (100)	100	-	-
21	0	0	30 (100)	0	0	30 (100)	100	-	-
22	0	0	30 (100)	0)	0	30 (100)	100	-	-
23	27 (90,0)	3 (10,0)	0	25 (83,3)	5 (16,7)	0	93,333	0,714	>0,001
24	0	30 (100)	0	0	30 (100)	0	100	-	-
25	14 (46,7)	16 (53,3)	0	15 (50,0)	15 (50,0)	0	96,667	0,933	>0,001
26	1 (3,3)	29 (96,7)	0	1 (3,3)	29 (96,7)	0	100	-	-
27	3 (10,0)	27 (90,0)	0	3 (10,0)	27 (90,0)	0	100	-	-
28	27 (90,0)	3 (10,0)	0	28 (93,3)	2 (6,7)	0	96,667	0,783	>0,001

Fonte: Elaborada pela autora, 2020.
Nota: Resultados expressos por n (%).

7 DISCUSSÃO

Realizou-se a adaptação cultural do instrumento *WHO Surgical Safety Checklist: for Radiological Interventions ONLY* para disponibilizar seu uso no Brasil. O instrumento foi selecionado visto que não há *checklist* específico para serviços de radiologia intervencionista validado para a realidade brasileira.

Os *checklists* em saúde são projetados para incentivar uma discussão clara, rápida e objetiva, em momentos estratégicos do procedimento, para a captura das principais informações (SCOTT e SHAFI, 2018).

Evidências demonstram que o *checklist* contribui positivamente em diminuições de complicações em assistência de saúde (GLOBALSURG COLLABORATIVE, 2016). Uma lista de verificação reduz a dependência de memória e estabelece um mecanismo para verificar elementos que poderiam ser esquecidos, devido a tendências humanas (SINGHAL e UTHAPPA, 2019).

Os pacientes sentem a ferramenta como positiva para seu sentimento de segurança durante o procedimento e não se sentem desconfortáveis pela reconfirmação de itens, como identidade, tipo e localização do procedimento (BERGS *et al*., 2018).

Em outros países, embora ainda com poucas publicações, a utilização do *checklist* para radiologia intervencionista tem se mostrado positiva (RAFIEI *et al*., 2016; HAWRANEK *et al*., 2015). Ele proporciona à equipe um suporte em comunicação, auxiliando na assistência segura ao paciente (CAHILL *et al*., 2015). Um dos pontos positivos da ferramenta é a acessibilidade, a facilidade e a praticidade de execução, podendo auxiliar na abertura de um canal de comunicação entre a equipe multiprofissional (GORDON *et al*., 2014).

Em 2012 e, novamente, em 2016 o RCR auditou o uso da ferramenta *WHO Surgical Safety Checklist: for Radiological Interventions ONLY* em diversas modalidades e subespecialidades de serviços de intervenção radiológica no Reino Unido. Em 2012, 93% das instituições implementaram total ou parcialmente o *checklist*. Em 2016, houve melhora, com 98% das instituições implementando a ferramenta, sendo que 48% a utilizavam para todos procedimentos em todas as modalidades, 50% para alguns procedimentos e 2% não a utilizavam. As instituições auditadas informaram que o processo é

geralmente percebido como eficaz para a segurança do paciente. As principais limitações para a implantação da ferramenta apontadas foram: o instrumento não é apropriado para procedimentos menores; falta de comprometimento da equipe; e o fato da lista de verificação ser muito longa e conter alguns dados desnecessários (ARIYANAYAGAM *et al*., 2019).

Hawranek *et al*. (2015), em estudo realizado na Polônia, analisaram o efeito do *checklist* na diminuição de eventos adversos em procedimentos cardiológicos invasivos e eletrofisiológicos. O estudo foi realizado com 2.064 pacientes, sendo 1.011 (grupo controle) pacientes atendidos sem a utilização do *checklist* e 1.053 (grupo intervenção) pacientes atendidos com a utilização do *checklist*. A utilização do *checklist* associou-se a uma significativa redução de eventos adversos, principalmente sangramentos (3,0% vs. 0,9%; p = 0,001), diminuição de erros relacionados à assistência à saúde e contribuição positiva na organização e na comunicação entre a equipe, proporcionando melhor qualidade da assistência prestada ao paciente.

Rafiei *et al*. (2016) descreveram itens potenciais a comporem um *checklist* para procedimentos radiológicos invasivos e ressaltaram que a implementação de uma ferramenta neste sentido requer *design* cuidadoso, implementação eficaz, trabalho em equipe e envolvimento da gestão. Destacaram, ainda, que a lista de verificação pré-procedimento não é uma panaceia, mas projetada para promover a comunicação e favorecer o trabalho em equipe, em um esforço mútuo para garantir a segurança do paciente (RAFIEI *et al*., 2016).

No entanto, outras pesquisas não apresentam significância estatística na diminuição de eventos adversos com o uso do *checklist*. Estudo realizado em serviço de cateterismo de cardiopatia congênita objetivou avaliar o impacto de uma lista de verificação em complicações, nas atitudes e percepções dos profissionais em relação à segurança e ao senso de trabalho em equipe. Para tanto, avaliaram-se dados de 741 paciente, sendo 371 avaliações de complicações de forma prospectiva (antes da implementação do *checklist*) e 370 após a implementação do *checklist*. Os autores concluíram que não houve diferença significativa no clima de segurança e nem nas taxas de complicações, no entanto a equipe referiu maior conscientização com o uso da lista de verificação (GORDON *et al*., 2014).

Munn *et al*. (2018), com objetivo de avaliar a utilização do *checklist* e a percepção dos radiologistas sobre o instrumento, realizaram estudo em quatro serviços de radiologia intervencionista situados em três estados da Austrália, e todos utilizavam listas de verificações de segurança desenvolvidas localmente. Os autores realizaram observações diretas durante 39 procedimentos radiológicos, avaliação retrospectiva (12 meses antes de iniciarem as observações) em amostra aleatória de 137 prontuários e discussão em grupo focal via teleconferência com quatro radiologistas responsáveis pela lista de verificação. Os resultados apresentaram média de preenchimento de apenas 38% dos itens da lista, sendo eles geralmente a confirmação do paciente, do procedimento e de alergias − discrepante da média de preenchimento registrada em prontuário que foi de 64%. Os radiologistas apontaram a falta de trabalho em equipe, a mentalidade empresarial com foco em velocidade e a presença de muitos itens nas listas de verificações como fatores limitadores para utilização da lista e sugeriram um coordenador responsável, o envolvimento de toda equipe e a possibilidade dos membros da equipe solicitarem uma pausa, caso seja verificado a necessidade.

Zhu *et al*. (2016) apontaram o *checklist* como uma das ferramentas de boas práticas em procedimentos de angiografia e intervenção transradial em Xangai. Para melhorar a adesão ao uso da ferramenta, os autores verificaram as barreiras para implementação do *checklist*, realizaram um planejamento estratégico, implementaram as medidas e, por fim, alcançaram o preenchimento do *checklist* em 78% dos procedimentos.

Barreiras como falta de conhecimento sobre o *checklist* e seu preenchimento; falta de liderança − nenhum membro da equipe responsável em promover e auditar o *checklist*; equipe considerar consumo de tempo e burocracia adicional; procedimentos fora do expediente que envolvem funcionários de outros setores não familiarizados com a ferramenta e extravio dos instrumentos preenchidos podem contribuir para a pouca efetividade do instrumento. Neste sentido, o enfermeiro pode ser o profissional principal na conscientização, no treinamento, no engajamento e na auditoria na implantação da ferramenta (PUTTICK *et al*., 2016).

A comunicação disfuncional durante procedimentos assistenciais tem efeito negativo sobre o desempenho da equipe, a qualidade assistencial e a

segurança do paciente (GAROSI *et al*., 2019). As salas de procedimentos são historicamente hierárquicas, e isso se reflete no comportamento dos membros da equipe, dificultando o desenvolvimento de uma cultura de segurança (SCOTT e SHAFI, 2018). Nesse sentido, um *checklist* tem potencial para otimizar a comunicação, o trabalho e a cooperação entre a equipe, corroer barreiras hierárquicas contraproducentes para a qualidade do atendimento e antecipar problemas potenciais. Em estudo realizado com a aplicação de uma lista de verificação em procedimentos de neurointervenção, observou-se que a ferramenta colaborou para a comunicação significativamente melhorada e redução significativa no total de eventos adversos (FARGEN *et al*., 2013; SINGHAL e UTHAPPA, 2019).

Para que a implementação do *checklist* seja efetiva, é necessário que cada centro envolva a equipe e adapte o *checklist* à realidade e à logística do serviço (CAHILL *et al*., 2015).

Nos modelos de *checklist* de segurança em serviços de radiologia intervencionista propostos na literatura, observamos a frequente preocupação em verificar apresentação da equipe, histórico, consentimento informado, revisão de imagens prévias, sedação e analgesia, função renal, *status* de anticoagulação, alergias, terapia prévia com heparina, preocupações com equipamentos, instruções pós-procedimento e anotações realizadas (CAHILL *et al*., 2015; GORDON *et al*., 2014; HAWRANEK *et al*., 2015; PUTTICK *et al*., 2016; SINGHAL e UTHAPPA, 2019).

Zhu (2016) destaca a importância de ser incluída no *checklist* a avaliação da perviedade arterial. Puttick *et al*. (2016), em sua adaptação do *checklist* de segurança em radiologia intervencionista, removeram a seção sobre anestesia geral, considerando o baixo índice de pacientes submetidos a este procedimento. Outros estudos que sugeriram modelos de *checklist* também optaram por não incluir a seção específica para anestesia geral no instrumento (CAHILL *et al*., 2015; HAWRANEK *et al*., 2015; SINGHAL e UTHAPPA, 2019).

Gordon (2014), em sua proposta, não fez divisão específica para casos de anestesia geral, mas manteve as questões: "*Difficult airway or aspiration risk? Yes/No, and equipment/assistance available*" e "*Are there any patient-specific concerns?*" para serem checadas em todos os pacientes (CAHILL *et al*., 2015).

Não é objetivo das listas de verificações substituírem os protocolos de boas práticas clínicas ou abrangerem todas as possibilidades de erros no serviço, mas proporcionarem uma pausa para reflexão e discussão antes da realização de qualquer procedimento invasivo (WONG *et al*., 2015).

Em radiologia intervencionista, estudo maiores e multicêntricos são necessários para verificar a efetividade da utilização de uma lista de verificações e sua correlação com a diminuição de complicações e mortalidade (FARGEN *et al*., 2013; PUTTICK *et al*., 2016; RAFIEI *et al*., 2016).

Em 2019, o RCR publicou uma versão atualizada do *WHO Surgical Safety Checklist: for Radiological Interventions ONLY* (Anexo E), entretanto as modificações foram apenas no layout do intrumento (THE ROYAL COLLEGE OF RADIOLOGISTS, 2019).

8 CONCLUSÃO

O processo de adaptação cultural e a validação do instrumento *WHO Surgical Safety Checklist: for Radiological Interventions ONLY* resultou no *WHO Surgical Safety Checklist: for Radiological Interventions ONLY* Versão Português-Brasileiro. O instrumento possui 28 itens a serem conferidos em três diferentes momentos do procedimento: *sign in*, antes do paciente ser anestesiado (seja anestesia geral ou regional); *time out*, antes do início do procedimento, apenas em caso de anestesia geral; e *sign out,* antes que qualquer membro da equipe saia da sala.

A tradução, adaptação cultural e validação face e conteúdo do *WHO Surgical Safety Checklist: for Radiological Interventions ONLY* satisfez os critérios de equivalência entre o instrumento original e o traduzido. O instrumento mostrou-se compreensível e viável podendo ser aplicado em procedimentos radiológicos invasivos no Brasil.

O WHO Surgical Safety Checklist: for Radiological Interventions ONLY encontra-se disponível apenas na versão em inglês. Sua adaptação para o português amplia seu uso no contexto latino-americano. Ressalta-se a importância de se realizarem estudos futuros que avaliem a aplicação do instrumento, para verificar a necessidade de readequação da versão aqui disponibilizada.

REFERÊNCIAS

AGUIAR, B. F. *et al*. Importância dos cuidados de enfermagem no cateterismo cardíaco. **Ciência, Cuidado e Saúde**, v. 15, n. 3, p. 460, jul. 2016. Disponível em: http://www.periodicos.uem.br/ojs/index.php/CiencCuidSaude/article/view/24894/18293. Acesso em: 2 jan. 2020.

ALPENDRE, F. T. *et al*. Cirurgia segura: validação de checklist pré e pós-operatório. **Revista Latino-Americana de Enfermagem**, v. 25, n. e2907, jul. 2017. Disponível em: http://www.scielo.br/scielo.php?script=sci_arttext&pid=S0104-11692017000100357&lng=en&nrm=iso. Acesso em: 2 jan. 2020.

ANDERSON, D. J. *et al*. Strategies to Prevent Surgical Site Infections in Acute Care Hospitals: 2014 Update. **Infection Control & Hospital Epidemiology**, v. 35, n. 6, p. 605-627, jun. 2014. Disponível em: https://www.ncbi.nlm.nih.gov/pmc/articles/PMC4267723/pdf/nihms-638942.pdf. Acesso em: 2 jan. 2020.

ANDERSON, J.; ABRAHAMSON, K. Your Health Care May Kill You: Medical Errors. **Studies in Health Technology and Informatics**, p. 13-17, 2017. Disponível em: http://ebooks.iospress.nl/publication/46132. Acesso em: 2 jan. 2020.

ANDRADE, L. S. DE *et al*. Surgical Site Infection Prevention Bundle in Cardiac Surgery. **Arquivos Brasileiros de Cardiologia**, v.112, n. 6, p. 769-774, 2019. Disponível em: http://www.scielo.br/pdf/abc/v112n6/pt_0066-782X-abc-20190070.pdf. Acesso em: 2 jan. 2020.

ANGLE, J. F. *et al*. Quality Improvement Guidelines for Preventing Wrong Site, Wrong Procedure, and Wrong Person Errors: Application of the Joint Commission "Universal Protocol for Preventing Wrong Site, Wrong Procedure, Wrong Person Surgery" to the Practice of Interventional Radiology. **Journal of Vascular and Interventional Radiology**, v. 19, n. 8, p. 1145-1151, ago. 2008. Disponível em: https://www.jvir.org/article/S1051-0443(08)00473-9/pdf. Acesso em: 2 jan. 2020.

ARIYANAYAGAM, T. *et al*. UK national audit of safety checks for radiology interventions. **The British Journal of Radiology,** v. 92, n. 1094, p. 20180637,

fev. 2019. Disponível em: https://www.birpublications.org/doi/full/10.1259/bjr.20180637. Acesso em: 2 jan. 2020.

BARBOSA, M. H. *et al*. Complicações em pacientes submetidos à Angioplastia Coronariana Transluminal Percutânea. **Enfermería Global**, n. 31, p. 24-33, jul. 2013. Disponível em: http://scielo.isciii.es/pdf/eg/v12n31/pt_clinica2.pdf. Acesso em: 2 jan. 2020.

BATHLA, S. *et al*. Which pen? A comparative study of surgical site markers. **Journal of Perioperative Practice,** v. 28, n. 1-2, p. 21-26, jan. 2018. Disponível em: https://journals.sagepub.com/doi/pdf/10.1177/1750458917742049. Acesso em: 2 jan. 2020.

BERGS, J. *et al*. Seen through the patients' eyes: surgical safety and checklists. **International Journal for Quality in Health Care,** v. 30, n. 2, p. 118-123, mar. 2018. Disponível em: https://academic.oup.com/intqhc/article-abstract/30/2/118/4797520. Acesso em: 2 jan. 2020.

BOSCÁ-MAYANS, M. DEL R.; ARANA, E.; SÁNCHEZ-APARISI, E. Diseño de una lista de verificación para radiología intervencionista. **Enfermería Clínica,** v. 22, n. 6, p. 299-303, nov. 2012. Disponível em: https://www.elsevier.es/es-revista-enfermeria-clinica-35-articulo-diseno-una-lista-verificacion-radiologia-S1130862112001325. Acesso em: 2 jan. 2020.

BRITISH INSTITUTE OF RADIOLOGY, SOCIETY AND COLLEGE OF RADIOGRAPHERS AND THE ROYAL COLEEGE OG RADIOLOGISTS. A guide to understanding the implication of the Ionising Radiation (Medical Exposure) Regulationd in diagnostic and interventional radiology. London: **The Royal College of Radiologists**, 2015. Disponível em: https://www.rcr.ac.uk/sites/default/files/bfcr152_irmer.pdf. Acesso em: 2 jan. 2020.

CAHILL, T. J. *et al*. A patient safety checklist for the cardiac catheterisation laboratory. **Heart**, v. 101, n. 2, p. 91-93, 15 jan. 2015. Disponível em: https://www.ncbi.nlm.nih.gov/pubmed/25480884. Acesso em: 2 jan. 2020.

CHAWLA, R. *et al*. Race and contrast-induced nephropathy in patients undergoing coronaryangiography and cardiac catheterization. **International**

Journal of Cardiology. v. 230, n. 1, p. 610-613, mar. 2017. Disponível em: https://internationaljournalofcardiology.com/article/S0167-5273(16)34534-X/fulltext. Acesso em: 2 jan. 2020.

CHEHAB, M. A. *et al*. Adult and Pediatric Antibiotic Prophylaxis during Vascular and IR Procedures: A Society of Interventional Radiology Practice Parameter Update Endorsed by the Cardiovascular and Interventional Radiological Society of Europe and the Canadian Association for Interventional Radiology. **Journal of Vascular and Interventional Radiology**, v. 29, n. 11, p. 1483-1501.e2, nov. 2018. Disponível em: https://www.jvir.org/article/S1051-0443(18)31259-4/pdf. Acesso em: 2 jan. 2020.

CORNELIS, F. H. *et al*. Sedation and analgesia in interventional radiology: Where do we stand, where are we heading and why does it matter? **Diagnostic and Interventional Imaging**, v. 100, n. 12, p. 753-762, dez. 2019. Disponível em: https://www.sciencedirect.com/science/article/pii/S2211568419302293. Acesso em: 2 jan. 2020.

CORSO, R. *et al*. Use of “Time-Out” checklist in interventional radiology procedures as a tool to enhance patient safety. **La Radiologia Medica**, v. 119, n. 11, p. 828–834, nov. 2014. Disponível em: https://link.springer.com/article/10.1007%2Fs11547-014-0397-9. Acesso em: 2 jan. 2020.

DANTAS, R. A. S. **Adaptação cultural e validação do questionário de senso de coerência de Antonovsky em uma amostra de pacientes cardíacos brasileiros**. Tese (Livre-Docência). 115f. Ribeirão Preto: Escola de Enfermagem de Ribeirão Preto da USP, 2007. Disponível em: http://www.uc.pt/en/fmuc/phdhs/Courses/HealthandDevelopment/LIVRE-DOCA_NCIA_ROSANA_APARECIDA_SPADOTI_DANTAS_1_.pdf. Acesso em: 2 jan. 2020.

DIEGO, L. A. DOS S. *et al*. Construção de uma ferramenta para medida de percepções sobre o uso do checklist do Programa de Cirurgia Segura da Organização Mundial da Saúde. **Revista Brasileira de Anestesiologia,** v. 66, n. 4, p. 351-355, jul. 2016. Disponível em: http://www.scielo.br/scielo.php?script=sci_arttext&pid=S0034-70942016000400351. Acesso em: 2 jan. 2020.

DUARTE, S. da C. M. *et al*. Eventos adversos e segurança na assistência de enfermagem. **Revista Brasileira de Enfermagem,** v. 68, n. 1, p. 144–154, fev.

2015. Disponível em: http://www.scielo.br/pdf/reben/v68n1/0034-7167-reben-68-01-0144.pdf. Acesso em: 2 jan. 2020.

FARGEN, K. M. *et al*. Enhanced staff communication and reduced near-miss errors with a neurointerventional procedural checklist. **Journal of NeuroInterventional Surgery,** v. 5, n. 5, p. 497–500, set. 2013. Disponível em: https://jnis.bmj.com/content/5/5/497.long. Acesso em: 2 jan. 2020.

FERRER, M. *et al*. Validity and reliability of the St George's Respiratory Questionnaire after adaptation to a different language and culture: the Spanish example. **The European Respiratory Journal,** v. 9, n. 6, p. 1160-1166, 1996.. Disponível em: http://taiss.com/gepoc/gecarp/gepubli/publdoc/032-036-ferrerm-validity-reliability-st-georges-respiratory-questionnaire.pdf. Acesso em: 2 jan. 2020.

GAROSI, E. *et al*. Concerns About Verbal Communication in the Operating Room: A Field Study. **Human Factors: The Journal of the Human Factors and Ergonomics Society**, p. 001872081985827, jul. 2019. Disponível em: https://journals.sagepub.com/doi/abs/10.1177/0018720819858274?rfr_dat=cr_pub%3Dpubmed&url_ver=Z39.88-2003&rfr_id=ori%3Arid%3Acrossref.org&journalCode=hfsa. Acesso em: 2 jan. 2020.

GEORGE, S. *et al*. Percutaneous coronary intervention and bleeding complications. **European Medical Journal,** v. 4, n. 1, p. 100-109, jun. 2016. Disponível em: https://emj.emg-health.com/wp-content/uploads/sites/2/2016/06/Percutaneous-Coronary-Intervention-and-Bleeding-Complications.pdf. Acesso em: 2 jan. 2020.

GLOBALSURG COLLABORATIVE. Mortality of emergency abdominal surgery in high-, middle- and low-income countries: GlobalSurg Collaborative. **British Journal of Surgery**, v. 103, n. 8, p. 971-988, jul. 2016. Disponível em: https://bjssjournals.onlinelibrary.wiley.com/doi/full/10.1002/bjs.10151. Acesso em: 2 jan. 2020.

GORDON, B. M. *et al*. Utility of Preprocedure Checklists in the Congenital Cardiac Catheterization Laboratory: Checklists for Cardiac Catheterization. **Congenital Heart Disease**, v. 9, n. 2, p. 131-137, mar. 2014. Disponível em: https://www.ncbi.nlm.nih.gov/pubmed/23750802. Acesso em: 2 jan. 2020.

GUILLEMIN, F.; BOMBARDIER, C.; BEATON, D. Cross-cultural adaptation of health-related quality of life measures: Literature review and proposed guidelines. **Journal of Clinical Epidemiology**, v. 46, n. 12, p. 1417-1432, dez. 1993. Disponível em: https://www.jclinepi.com/article/0895-4356(93)90142-N/pdf. Acesso em: 2 jan. 2020.

HAUGEN, A. S. *et al*. Effect of the World Health Organization Checklist on Patient Outcomes: A Stepped Wedge Cluster Randomized Controlled Trial. **Annals of Surgery,** v. 261, n. 5, p. 821-828, mai. 2015. Disponível em: https://insights.ovid.com/pubmed?pmid=24824415. Acesso em: 2 jan. 2020.

HAWRANEK, M. *et al*. Periprocedural checklist in the catheterisation laboratory is associated with decreased rate of treatment complications. **Kardiologia Polska,** v. 73, n. 7, p. 511-519, 2015. Disponível em: https://www.mp.pl/kardiologiapolska/en/node/14320/pdf. Acesso em: 2 jan. 2020.

HAYNES, A. B. *et al*. A Surgical Safety Checklist to Reduce Morbidity and Mortality in a Global Population. **New England Journal of Medicine,** v. 360, n. 5, p. 491-499, 29 jan. 2009. Disponível em: https://www.nejm.org/doi/full/10.1056/NEJMsa0810119. Acesso em: 2 jan. 2020.

HOSPITAL DE CLÍNICAS DA UNIVERSIDADE FEDERAL DO TRIÂNGULO MINEIRO (HC/UFTM). **Hospital de Clínicas: Nossa História**. 2016. Disponivel em: http://www.ebserh.gov.br/web/hc-uftm/historia. Acesso em: 2 jan. 2020.

KOETSER, I. C. J. *et al*. A Checklist to Improve Patient Safety in Interventional Radiology. **CardioVascular and Interventional Radiology,** v. 36, n. 2, p. 312-319, abr. 2013. Disponível em: https://www.ncbi.nlm.nih.gov/pmc/articles/PMC3595473/pdf/270_2012_Article_395.pdf. Acesso em: 2 jan. 2020.

KORKIAKANGAS, T. Mobilising a team for the WHO Surgical Safety Checklist: a qualitative video study. **BMJ Quality & Safety,** v. 26, n. 3, p. 177-188, mar. 2017. Disponível em: https://qualitysafety.bmj.com/content/26/3/177.long. Acesso em: 2 jan. 2020.

LEAPE, L. L. The Checklist Conundrum. **New England Journal of Medicine.** v. 370, n. 11, p. 1063–1064, 13 mar. 2014. Disponível em: https://www.nejm.org/doi/full/10.1056/NEJMe1315851. Acesso em: 2 jan. 2020.

LEE, M. J. *et al*. Patient Safety in Interventional Radiology: A CIRSE IR Checklist. **CardioVascular and Interventional Radiology.** v. 35, n. 2, p. 244–246, abr. 2012. Disponível em: https://www.ncbi.nlm.nih.gov/pmc/articles/PMC3298647/pdf/270_2011_Article_289.pdf. Acesso em: 2 jan. 2020.

MAIA, C. S. *et al*. Notificações de eventos adversos relacionados com a assistência à saúde que levaram a óbitos no Brasil, 2014-2016. **Epidemiologia e Serviços de Saúde**, v. 27, n. 2, mai. 2018. Disponível em: http://www.scielo.br/pdf/ress/v27n2/2237-9622-ress-27-02-e2017320.pdf. Acesso em: 2 jan. 2020.

MAKARY, M. A.; DANIEL, M. Medical error—the third leading cause of death in the US. **The British Medical Journal**, 353, p. i2139, 3 mai. 2016. Disponível em: https://www.bmj.com/content/353/bmj.i2139. Acesso em: 2 jan. 2020.

MAZIERO, E. C. S. Adesão ao uso de um checklist cirúrgico para segurança do paciente. **Revista Gaúcha de Enfermagem***,* v. 36, n. 4, p. 14-20, dez. 2015. Disponível em: http://www.scielo.br/pdf/rgenf/v36n4/pt_1983-1447-rgenf-36-04-00014.pdf. Acesso em: 2 jan. 2020.

MOURA, R.; BACCHIM NETO, F. A. Proteção radiológica aplicada à radiologia intervencionista. **Jornal Vascular Brasileiro,** v. 14, n. 3, p. 197–199, set. 2015. Disponível em: http://www.scielo.br/pdf/jvb/v14n3/1677-5449-jvb-14-3-197.pdf. Acesso em: 2 jan. 2020.

MUNN, Z. *et al*. Mixed methods study on the use of and attitudes towards safety checklists in interventional radiology. **Journal of Medical Imaging and Radiation Oncology,** v. 62, n. 1, p. 32–38, fev. 2018. Disponível em: https://onlinelibrary.wiley.com/doi/pdf/10.1111/1754-9485.12633. Acesso em: 2 jan. 2020.

PATEL, I. J. *et al*. Society of Interventional Radiology Consensus Guidelines for the Periprocedural Management of Thrombotic and Bleeding Risk in Patients Undergoing Percutaneous Image-Guided Interventions—Part II: Recommendations. **Journal of Vascular and Interventional Radiology**, v. 30,

n. 8, p. 1168-1184.e1, ago. 2019. Disponível em: https://www.jvir.org/article/S1051-0443(19)30407-5/pdf. Acesso em: 2 jan. 2020.

POLIT, D. F.; BECK, C. T. **Fundamentos de Pesquisa em Enfermagem** - Avaliação de Evidências para a Prática da Enfermagem. Tradução de Denise Regina de Sales. 9. ed. Philadelphia: Wolters Kluwer Health, 2018.

PUTTICK, T. *et al*. Barriers to a safety checklist and methods to improve usage of the WHO safety checklist in interventional radiology. **British Journal of Radiology**, v. 2, n. 2, p. 20150128, mai. 2016. Disponível em: https://www.ncbi.nlm.nih.gov/pmc/articles/PMC6180857/pdf/bjrcr.20150128.pdf. Acesso em: 2 jan. 2020.

RAFIEI, P. *et al*. Society of Interventional Radiology IR Pre-Procedure Patient Safety Checklist by the Safety and Health Committee. **Journal of Vascular and Interventional Radiology**, v. 27, n. 5, p. 695-699, maio 2016. Disponível em: https://www.jvir.org/article/S1051-0443(16)00390-0/pdf. Acesso em: 2 jan. 2020.

RIPLEY, B. A. et al. Improving the Informed Consent Conversation: A Standardized Checklist that Is Patient Centered, Quality Driven, and Legally Sound. **Journal of Vascular and Interventional Radiology**, v. 26, n. 11, p. 1639–1646, nov. 2015. Disponível em: https://www.jvir.org/article/S1051-0443(15)00549-7/pdf. Acesso em: 2 jan. 2020.

SAINT-EXUPÉRY, A. **O pequeno príncipe**. Tradução de Dom Marcos Barbosa. Rio de Janeiro: Agir, 2009.

SCOTT, D.; SHAFI, M. The WHO surgical checklist: Improving safety in our operating theatres. **Obstetrics, Gynaecology & Reproductive Medicine**, v. 28, n. 9, p. 295-297, set. 2018. Disponível em: https://www.obstetrics-gynaecology-journal.com/article/S1751-7214(18)30128-3/pdf. Acesso em: 2 jan. 2020.

SINGHAL, S.; UTHAPPA, M. C. Role of a Checklist to Improve Patient Safety in Interventional Radiology. **Journal of Clinical Interventional Radiology ISVIR**, v. 3, p. 157-161, jul. 2019. Disponível em: https://www.thieme-connect.com/products/ejournals/pdf/10.1055/s-0039-1693536.pdf

TASE, T. H.; QUADRADO, E. R. S.; TRONCHIN, D. M. R. Avaliação do risco de erro na identificação de mulheres numa maternidade pública. **Revista Brasileira de Enfermagem**, v. 71, n. 1, p. 120-125, fev. 2018. Disponível em: http://www.scielo.br/pdf/reben/v71n1/pt_0034-7167-reben-71-01-0120.pdf. Acesso em: 2 jan. 2020.

THE AMERICAN SOCIETY OF ANESTHESIOLOGISTS. Practice Guidelines for Moderate Procedural Sedation and Analgesia 2018: A Report by the American Society of Anesthesiologists Task Force on Moderate Procedural Sedation and Analgesia, the American Association of Oral and Maxillofacial Surgeons, American College of Radiology, American Dental Association, American Society of Dentist Anesthesiologists, and Society of Interventional Radiology. **Anesthesiology.** v. 128, n. 3, p. 437-479, mar. 2018. Disponível em: https://static1.squarespace.com/static/54d14bfce4b02b4744e70d6d/t/5a8708ad24a6943b16cb9ea2/1518799460707/ASA+Practice+Guidelines+Moderate+Procedural+Sedation_2018.pdf. Acesso em: 2 jan. 2020.

THE ROYAL COLLEGE OF RADIOLOGISTS. **Guidance on implementing safety checklists for radiological procedures, second edition**. London: The Royal College of Radiologists, 2019. Disponível em: https://www.rcr.ac.uk/system/files/publication/field_publication_files/bfcr191_checklists-radiological-procedures.pdf. Acesso em: 2 jan. 2020.

______. **Guidance for Fellows in implementing surgical safety checklist for radiological procedures**. London: The Royal College of Radiologists, fev. 2013. Disponível em: https://www.rcr.ac.uk/publication/guidance-fellows-implementing-surgical-safety-checklists-radiological-procedures. Acesso em: 2 jan. 2020.

______. **Standards for the NPSA and RCP safety checklist for radiological interventions**. London: The Royal College of Radiologists, dez. 2010. Disponível em: https://www.bsir.org/media/resources/NPSA_RCR_checklist_RCR_2010.pdf. Acesso em: 2 jan. 2020.

______. Guidelines for radiologists in implementing the NPSA Safe Surgery requirement. **The Royal College of Radiologists**, London, fev. 2009. Disponível em: https://www.bsir.org/media/resources/NPSA_checklist_guidance_RCR_2009.pdf. Acesso em: 2 jan. 2020.

URBACH, D. R. *et al*. Introduction of Surgical Safety Checklists in Ontario, Canada. **New England Journal of Medicine**, v. 370, n. 11, p. 1029-1038, 13 mar. 2014. Disponível em: https://www.nejm.org/doi/full/10.1056/NEJMsa1308261. Acesso em: 2 jan. 2020.

URMAN, R. D. *et al.* Impact of Respiratory Compromise in Inpatient Interventional Radiology Procedures with Moderate Sedation in the United States. **Radiology**, v. 292, n. 3, p. 702-710, set. 2019. Disponível em: https://pubs.rsna.org/doi/pdf/10.1148/radiol.2019182455. Acesso em: 2 jan. 2020.

VAN KLEI, W. A. *et al*. Effects of the Introduction of the WHO "Surgical Safety Checklist" on In-Hospital Mortality: A Cohort Study. **Annals of Surgery**, v. 255, n. 1, p. 44–49, jan. 2012. Disponível em: https://insights.ovid.com/pubmed?pmid=22123159. Acesso em: 2 jan. 2020.

WONG, S. S. N. *et al*. Impact and Culture Change After the Implementation of a Preprocedural Checklist in an Interventional Radiology Department. **Journal of Patient Safety,** p. 1, jul. 2015. Disponível em: https://insights.ovid.com/crossref?an=01209203-201912000-00025. Acesso em: 2 jan. 2020.

WORLD HEALTH ORGANIZATION (WHO). **WHO guidelines for safe surgery**. Geneva: WHO, 2009. Disponível em: https://apps.who.int/iris/bitstream/handle/10665/44185/9789241598552_eng.pdf;jsessionid=ACE327152331F30F85B67DCB4F46954C?sequence=1. Acesso em: 2 jan. 2020.

ZHU, L. *et al*. Nursing care of transradial angiography and intervention in a tertiary hospital in Shanghai: a best practice implementation project. **JBI Database of Systematic Reviews and Implementation Reports,** v. 14, n. 5, p. 286–294, maio 2016. Disponível em: https://insights.ovid.com/crossref?an=01938924-201605000-00018. Acesso em: 2 jan. 2020.

ANEXO A – *WHO Surgical Safety Checklist: for Radiological Interventions ONLY.*

WHO Surgical Safety Checklist: for Radiological Interventions ONLY

(adapted from the WHO Surgical Safety Checklist)

THE ROYAL COLLEGE OF RADIOLOGISTS

NHS
National Patient Safety Agency
National Reporting and Learning Service

SIGN IN (To be read out loud)

Before giving anaesthetic (local or general)

Have all team members introduced themselves by name and role?
- ☐ Yes

All team members verbally confirm:
- ☐ What is the patient's name?
- ☐ What procedure, site and position are planned?

If general anaesthetic given the two questions above should be moved to the beginning of TIME OUT

Has the patient confirmed his/her identity, site, procedure and consent?
- ☐ Yes

Has essential imaging been reviewed?
- ☐ Yes ☐ N/A

Are all IRMER requirements met?
- ☐ Yes

Is the procedural site marked?
- ☐ Yes ☐ N/A

Is the anaesthesia machine/monitoring equipment and medication check complete?
- ☐ Yes ☐ N/A

Does the patient have a:
known allergy?
- ☐ No ☐ Yes

anticipated risk of >500ml blood loss (7ml/kg in children)?
- ☐ No ☐ Yes (and adequate IV access/fluids planned)

Have risk factors for bleeding and renal failure been checked?
- ☐ Yes ☐ N/A

Has Antibiotic prophylaxis been given?
- ☐ Yes ☐ N/A

Has VTE prophylaxis been undertaken?
- ☐ Yes ☐ N/A

Is the required equipment available and in date?
- ☐ Yes

Are there any critical or unexpected steps you want the team to know about?
- ☐ Yes ☐ N/A

ONLY IF GENERAL ANAESTHETIC IS GIVEN

TIME OUT (To be read out loud)

Before start of radiological intervention
(for example needle to skin)

Anticipated critical events

Anaesthetist (if present):
- ☐ Is the anaesthetic machine check complete?

Does the patient have a difficult airway/aspiration risk?
- ☐ Yes ☐ N/A

- ☐ Are there any patient-specific concerns?
- ☐ What is the patient's ASA grade?
- ☐ What monitoring equipment and other specific levels of support are required, for example blood?

Registered practitioner/HCA:
- ☐ Are there any equipment issues or concerns?

Has the surgical site infection (SSI) bundle been undertaken?
- ☐ Yes ☐ N/A
  - Antibiotic prophylaxis
  - Patient warming
  - Hair removal
  - Glycaemic control

SIGN OUT (To be read out loud)

Before any member of the team leaves the room

Registered Practitioner/HCA verbally confirms with the team:
- ☐ Has the name and side of the procedure been recorded?
- ☐ Have all pieces of invasive equipment used been accounted for?
- ☐ Have any implanted devices been recorded?
- ☐ Have the specimens been labelled (including with patient's name)?
- ☐ Have any equipment problems been identified that need to be addressed?

Radiologist, Anaesthetist and Registered Practitioner:
- ☐ Have the instructions for post procedural care for this patient been agreed?

Remember to scan onto CRIS
or record checklist has been undertaken

PATIENT DETAILS

Last name:
First name:
Date of birth:
NHS Number:*
Date of Procedure:

*If the NHS Number is not immediately available, a temporary number should be used until it is

The checklist is for Radiology Interventions ONLY

This modified checklist must not be used for other surgical procedures.

© National Patient Safety Agency 2010. Copyright and other intellectual property rights in this material belong to the NPSA and all rights are reserved. The NPSA authorises UK healthcare organisations to reproduce this material for educational and non-commercial use.

www.nrls.npsa.nhs.uk

Ref: 1121 March 2010

ANEXO B – Autorização da *National Patient Safety Agency* para utilização do instrumento.

PL 372312 Permissions [<AD78850>] MESTRADO ×

nhs.identity@dh.gsi.gov.uk <nhs.identity@dh.gsi.gov.uk>
para eu

qua, 24 de ago de 2016 08:55

inglês > português Traduzir mensagem Desativar para: inglês

Good Afternoon Flavia

Further to your request for permission to use "Who Surgical Safety Checklist: for Radiological Intervencions ONLY".

The following section of the NHS England website explains how you may use content from the NHS England website and the contact details if you have further queries.

https://www.england.nhs.uk/terms-and-conditions/#copyright

Best regards

Margaret Cox
NHS Brand and Identity Helpline
nhs.identity@dh.gsi.gov.uk
Tel: 020 7972 5250
www.nhsidentity.nhs.uk

____

Original Message:
From: brandwebsite@nhsidentity.nhs.uk
Sent: 21:26:36
To: nhs.identity@dh.gsi.gov.uk
Subject: Contact Us

ANEXO C – Autorização do Comitê de Ética em Pesquisa

UFTM - HOSPITAL DAS CLÍNICAS DA UNIVERSIDADE FEDERAL DO TRIÂNGULO

PARECER CONSUBSTANCIADO DO CEP

DADOS DO PROJETO DE PESQUISA

Título da Pesquisa: Adaptação cultural e validação do instrumento WHO surgical safety checklist: for radiological intervencions ONLY: versão para o português brasileiro

Pesquisador: Maria Helena Barbosa

Área Temática:

Versão: 2

CAAE: 03781118.6.0000.8667

Instituição Proponente: Hospital de Clínicas da Universidade Federal do Triângulo Mineiro

Patrocinador Principal: Financiamento Próprio

DADOS DO PARECER

Número do Parecer: 3.109.638

Apresentação do Projeto:

Segundo o projeto apresentado pelo(s) pesquisador(es) as discussões sobre segurança do paciente nas unidades hospitalares configuram uma tendência mundial (DUARTE et al., 2015). Em 2008, a World Health Organization (WHO) publicou uma série de iniciativas para promover a segurança dos pacientes em procedimentos cirúrgicos. Sua campanha "Cirurgia Segura Salva Vidas" introduziu o conceito de um checklist, o Surgical Safety Checklist (SSC), destinado a identificar e controlar os riscos durante as três fases do procedimento cirúrgico: antes da indução da anestesia ("sign-in"), antes da incisão da pele ("time-out") e antes da saída da sala de operações ("sign-on") (MARJOT; MARUTHAPPU; SHALHOUB, 2013). O SSC é uma inovação simples que tem sido bem recebida pela equipe cirúrgica e sua utilização mostrou-se significativa na redução da morbimortalidade. O estudo pioneiro sobre a implementação do SSC, realizado por Haynes et al. (2009), avaliou a utilização desse checklist em oito hospitais com diversidades econômica, de localização e de sistemas de cuidados de saúde. Dados de 3.733 pacientes foram comparados, antes da introdução do checklist, com dados de 3.955 após a introdução do checklist, resultando na queda da taxa de mortalidade de 1,5% antes da introdução da lista de verificação para 0,8% após. A taxa de complicação caiu de 11 para 7% (HAYNES et al., 2009). Vários estudos subsequentes também evidenciaram reduções da mortalidade e morbidade com a utilização do SSC (HAUGEN et al., 2015; VAN KLEI et al., 2012). No entanto, nem todos os estudos suportam as conclusões anteriores, como evidenciado na pesquisa de Urbach et al. (2014), desenvolvida em Ontário, a qual não

relatou mudanças em termos de resultados pós-operatórios com a implementação regional do SSC. Esses achados discrepantes sugerem que a maneira pela qual os hospitais implementam o SSC é chave para sua efetividade. Em Ontário, 92 dos 101 hospitais estudados não modificaram o instrumento para atender suas necessidades (LEAPE, 2014). Para o sucesso da implementação do checklist é necessário que a equipe seja treinada e sensibilizada quanto a utilização do mesmo. Para melhorar a segurança do paciente, a implementação da lista de verificação exige cooperação entre anestesistas, cirurgiões e enfermeiros (KORKIAKANGAS, 2017). A WHO recomenda a adaptação do checklist de acordo com a realidade de cada instituição, abordagem essa que promove um sentido de apropriação do instrumento e melhor adesão ao checklist (WORLD HEALTH ORGANIZATION, 2008). A Radiologia Intervencionista (RI) é uma especialidade com incidência de complicações e de morbimortalidade menor em comparação aos procedimentos cirúrgicos por sua natureza minimamente invasiva (WONG et al., 2015). Entretanto, procedimentos de RI têm muitos aspectos em comum com operações cirúrgicas (complexidade, rápida resolução, urgência e emergência, trabalho em equipe, etc.) e, consequentemente, implicam em um risco potencial para falhas e complicações. Portanto, implementar uma lista de verificações em RI pode ter a mesma eficácia em segurança do paciente como os checklists cirúrgicos (CORSO; VACIRCA; PATELLI, 2014). Nesse sentido, a implantação de uma lista de verificação de segurança em RI poderia evitar muitas complicações e problemas potenciais (WONG et al., 2015). Os mesmos autores ressaltam que como um benefício secundário, o checklist em RI também pode servir como uma importante ferramenta de comunicação entre os membros da equipe. Em 2009, a National Patient Safety (NPSA) e o Royal College of Radiologists (RCR) adaptaram o checklist "Cirúrgica Segura" da WORLD HEALTH ORGANIZATION para um checklist específico para intervenções radiológicas para Inglaterra e países de Gales, intitulado "WHO Surgical Safety Checklist: for radiological Intervencional ONLY" (ROYAL COLLEGE OF RADIOLOGISTS, 2009) No Brasil, o checklist em cirurgias é uma prática em evolução (ALPENDRE et al., 2017; DIEGO et al., 2016; MAZIERO et al., 2015). No entanto, ainda não há publicação sobre a implantação de uma lista de verificações de segurança no serviço de hemodinâmica na literatura brasileira, instrumento já aplicado com êxito em outros países. O cuidado para não causar dano ao paciente é um dever dos profissionais de saúde. Assim, o checklist é uma ferramenta que pode auxiliar na segurança, prática clínica e na comunicação da equipe (RAFIEI et al., 2016). Frente ao exposto, é importante a condução do presente estudo com foco na validação do instrumento Who Surgical Safety Checklist: for Radiological Intervencions ONLY, instrumento que poderá ser aplicado no contexto brasileiro, tendo como benefícios a melhoria da segurança do paciente submetido a procedimentos radiológicos invasivos. Segundo o

(s) pesquisador(es), não é objetivo das listas de verificações substituírem os protocolos de boas práticas clínicas ou abrangerem todas as possibilidades de erros no serviço, mas sim proporcionar uma pausa para reflexão e discussão antes da realização de qualquer procedimento invasivo (WONG et al., 2015).

Objetivo da Pesquisa:
Segundo o(s) pesquisador(es), o objetivo é realizar a adaptação transcultural e a validação do instrumento WHO Surgical Safety Checklist: for Radiological Intervencions ONLY para o português brasileiro.

Avaliação dos Riscos e Benefícios:
Segundo o(s) pesquisador(es), a pesquisa apresenta risco de perda de confidencialidade dos dados, desta forma, os instrumentos de coleta de dados serão identificados por código numérico, garantindo-se o sigilo e o anonimato dos pacientes que participarão deste estudo. Os benefícios diretos para população estudada e a sociedade envolvem a utilização da versão brasileira do instrumento "WHO surgical safety checklist: for radiological intervencions ONLY" o que poderá contribuir para favorecer a segurança do paciente submetido a procedimentos na unidade de hemodinâmica. Os benefícios indiretos para população estudada e a sociedade relacionam-se com a possibilidade da implantação de uma lista de verificações nas unidades de procedimento na unidade de hemodinâmica.

Segundo os pesquisadores a pesquisa será suspensa ou encerrada caso: solicitado pelo Comitê de Ética em Pesquisa (HC-UFTM) ou pelo Setor que a permitiu (Hemodinâmica-HC/UFTM) e ao termino da pesquisa.

Comentários e Considerações sobre a Pesquisa:
Segundo os pesquisadores trata-se de pesquisa metodológica cuja proposta é a adaptação cultural e validação de um instrumento de segurança do paciente em procedimentos radiológicos invasivos.Esse tipo de pesquisa refere-se aos estudos sobre métodos de obtenção, organização e análise dos dados e trata da elaboração, validação e avaliação de instrumentos e técnicas de investigação (POLIT; BECK, 2018).
Segundo os pesquisadores o estudo será desenvolvido em uma unidade de hemodinâmica de um hospital público de ensino de grande porte. A escolha do campo de estudo baseou-se no critério de viabilidade de realização da pesquisa, por tratar-se de um hospital de ensino e apresentar duas

Endereço: R. Benjamin Constant, 16
Bairro: Nossa Srª da Abadia **CEP:** 38.025-470
UF: MG **Município:** UBERABA
Telefone: (34)3318-5319 **E-mail:** cep.hctm@ebserh.gov.br

salas de hemodinâmica com grande fluxo de procedimentos. O hospital não possui lista de verificações de segurança do paciente na unidade de hemodinâmica implantada em sua rotina.
Os critérios de inclusão para os juízes serão os seguintes:

a) brasileiros;
b) com domínio na língua inglesa;
c) doutorado;
d) 2 dos juízes devem possuir graduação em enfermagem e doutoramento, com reconhecido saber em qualidade e segurança do paciente.
e) 2 dos juízes devem possuir graduação em medicina, um anestesiologista atuante em hemodinâmica e outro hemodinamicista;
f) o quinto juiz deve ter formação em área de saúde, com título de doutor e reconhecido saber em estudos metodológicos com validação de instrumentos.
g) inserção em atividades de pesquisas na temática;
h) interesse expresso em colaborar com o estudo. Os dados relativos à titulação do profissional serão verificados a partir do Currículo Lattes.

O termo de consentimento livre e esclarecido (TCLE) para os juízes será obtido via e-mail pelo pesquisador responsável, após aprovação deste projeto nas devidas instâncias éticas.

Segundo os pesquisadores não será coletado termo de consentimento livre e esclarecido (TCLE) com pacientes visto que será realizada apenas observação do procedimento e feito anotações quanto à aplicabilidade do instrumento. A realização do procedimento é uma rotina no setor. Para avaliação pré-teste, a VPF será aplicada em uma amostra de 30 procedimentos, número recomendado por Beaton et al. (2007). Os resultados dessa etapa serão resumidos e submetidos ao grupo da pesquisa para revisão. Após análise, gerará a Versão Final (VF1). Durante a realização do procedimento, duas enfermeiras (pesquisador 1 e pesquisador 2) observarão e anotarão, de maneira independente e simultânea, se os itens contidos no instrumento VF1 foram conferidos pela equipe.

Considerações sobre os Termos de apresentação obrigatória:
Os pesquisadores apresentam:
- Autorização do Curso

- Projeto de pesquisa
- Check List do Projeto de Pesquisa
- Check List Documental
- Autorização da Gerência de Ensino e Pesquisa
- Termo de Responsabilidade do Pesquisador
- TCLE para os juízes
- Termo de Dispensa do TCLE para os pacientes
- Folha de Rosto
- Instrumento WHO surgical safety checklist: for radiological intervencions ONLY

Recomendações:

De acordo com as atribuições definidas na Resolução CNS 466/12 e norma operacional 001/2013, o colegiado do CEP-HC/UFTM manifesta-se pela aprovação do protocolo de pesquisa proposto.

Conclusões ou Pendências e Lista de Inadequações:

A aprovação do protocolo de pesquisa pelo CEP/UFTM dá-se em decorrência do atendimento à Resolução CNS 466/12 e norma operacional 001/2013, não implicando na qualidade científica do mesmo.

Conforme prevê a legislação, são responsabilidades, indelegáveis e indeclináveis, do pesquisador responsável, dentre outras: comunicar o início da pesquisa ao CEP; elaborar e apresentar os relatórios parciais (semestralmente) e final. Para isso deverá ser utilizada a opção 'notificação' disponível na Plataforma Brasil.

Considerações Finais a critério do CEP:

• O acompanhamento dos projetos na Plataforma Brasil é de inteira responsabilidade dos pesquisadores, não podendo ser alegado desconhecimento de pendências como justificativa para não cumprimento de prazos.

• A secretaria do CEP-HC/UFTM está à disposição para quaisquer esclarecimentos sobre trâmites e funcionalidades da Plataforma Brasil, durante os dias de segunda a sexta-feira, das 07:00 às 16:00 hrs. Telefone: 34 3318-5319. e-mail: cep.hctm@ebserh.gov.br.

Este parecer foi elaborado baseado nos documentos abaixo relacionados:

Tipo Documento	Arquivo	Postagem	Autor	Situação
Informações Básicas do Projeto	PB_INFORMAÇÕES_BÁSICAS_DO_PROJETO_1175594.pdf	17/12/2018 11:39:33		Aceito

Outros	INSTRUMENTO.pdf	17/12/2018 11:38:06	Maria Helena Barbosa	Aceito
Outros	CHECKLIST_DOCUMENTAL.pdf	17/12/2018 11:37:27	Maria Helena Barbosa	Aceito
TCLE / Termos de Assentimento / Justificativa de Ausência	TERMO_DISPENSA.pdf	17/12/2018 11:36:40	Maria Helena Barbosa	Aceito
Projeto Detalhado / Brochura Investigador	FORMULARIO_PROJETO.docx	17/12/2018 11:36:12	Maria Helena Barbosa	Aceito
Declaração de Pesquisadores	AUTORIZACAO_CURSO.pdf	30/11/2018 12:34:46	Maria Helena Barbosa	Aceito
Outros	CHECKLIST_PROJETO.pdf	27/11/2018 10:50:54	Maria Helena Barbosa	Aceito
Outros	AUTORIZACAO_GEP.pdf	27/11/2018 10:48:43	Maria Helena Barbosa	Aceito
Declaração de Pesquisadores	TERMO_PESQUISADOR.pdf	27/11/2018 10:47:28	Maria Helena Barbosa	Aceito
TCLE / Termos de Assentimento / Justificativa de Ausência	TCLE_COMITE_JUIZES.docx	27/11/2018 10:43:39	Maria Helena Barbosa	Aceito
Folha de Rosto	FOLHA_ROSTO.pdf	27/11/2018 10:19:09	Maria Helena Barbosa	Aceito

Situação do Parecer:
Aprovado

Necessita Apreciação da CONEP:
Não

UBERABA, 09 de Janeiro de 2019

Assinado por:
GILBERTO DE ARAUJO PEREIRA
(Coordenador(a))

Endereço: R. Benjamin Constant, 16
Bairro: Nossa Srª da Abadia **CEP:** 38.025-470
UF: MG **Município:** UBERABA
Telefone: (34)3318-5319 **E-mail:** cep.hctm@ebserh.gov.br

ANEXO D – Versão atualizada do *WHO Surgical Safety Checklist: for Radiological Interventions ONLY.*

Appendix 1.
Example of a World Health Organization (WHO) radiological interventions checklist

Before giving anaesthetic (local or general)

Have all team members introduced themselves by name and role?
☐ Yes

All team members verbally confirm:
☐ What is the patient's name?
☐ What proceduce, site and position are planned?
If general anaesthetic given the two questions above should be moved to the beginning of TIME OUT

Has the patient confirmed his/her identity, site, procedure and consent? ☐ Yes

Has essential imaging been reviewed? ☐ Yes

Are all IR(ME)R requirements met? ☐ Yes

Is the procedural site marked? ☐ Yes ☐ N/A

Is the anaesthesia machine/monitoring equipment and medication check complete? ☐ Yes ☐ N/A

Does the patient have a:
Known allergy? ☐ No ☐ Yes
Anticipated risk of >500 ml blood loss (7ml/kg in children)?
☐ No ☐ Yes (and adequate IV access/fluids planned)

Have risk factors for bleeding and renal failure been checked?
☐ Yes ☐ N/A

Has antibiotic prophylaxis been given? ☐ Yes ☐ N/A
Has VTE prophylaxis been undertaken? ☐ Yes ☐ N/A

Is the required equipment available and in date? ☐ Yes

Are there any critical or unexpected steps you want the team to know about? ☐ Yes ☐ N/A

ONLY IF GENERAL ANAESTHETIC IS GIVEN

TIME OUT (To be read out loud)

Before start of radiological intervention (for example needle to skin)

Anticipated critical events

Anaesthetist (if present):
☐ Is the anaesthetic machine check complete?

Does the patient have a difficult airway/ aspiration risk?
☐ Yes ☐ N/A
☐ Are there any patient-specific concerns?
☐ What is the patient's ASA grade?
☐ What monitoring equipment and other specific levels of support are required, for example blood?

Registered practitioner/HCA:
☐ Are there any equipment issues or concerns?

Has the surgical site infection (SSI) bundle been undertaken
☐ Yes ☐ N/A
- Antibiotic prophylaxis
- Patient warming
- Hair removal
- Glycaemic control

The checklist is for radiology interventions ONLY

This modified checklist must not be used for other surgical procedures

SIGN OUT (To be read out loud)

Before any member of the team leaves the room

Registered practitioner/HCA verbally confirms with the team:
☐ Has the name and side of the procedure been recorded?
☐ Have all the pieces of invasive equipment used been accounted for?
☐ Have any implanted devices been recorded?
☐ Have the specimens been labelled (including with the patient's name?
☐ Have any equipment problems been identified that need to be addressed?

Radiologist, anaesthetist and registered practitioner:
☐ Have the instructions for post-procedural care for this patient been agreed?

PATIENT DETAILS

Last name:	
First name:	
Date of birth:	
NHS number:*	
Date of procedure:	

*If the NHS number isn't immediately available, a temporary number should be used until it is

Remember to scan onto CRIS or record checklist has been undertaken

This checklist has been adapted from the WHO Surgical Safety checklist. © National Patient Safety Agency 2010. Copyright and other intellectual property rights in this material belong to the NPSA and all rights are reserved. The NPSA authorises UK healthcare organisations to reproduce the material for educational and non-commercial uses.

APÊNDICE A – Termo de Consentimento Livre e Esclarecido (TCLE) – comitê de juízes.

Universidade Federal do Triângulo Mineiro
Pós-graduação *Stricto Sensu* em Atenção à Saúde
Av. Getúlio Guaritá, 159 – Nossa Sra. da Abadia, Uberaba – MG, 38025-440

TERMO DE ESCLARECIMENTO

(Para participantes do Comitê de Juízes)

TÍTULO DA PESQUISA: **Adaptação cultural e validação do instrumento *WHO Surgical Safety Checklist: for Radiological Interventions ONLY*: versão para o português brasileiro**

Convidamos você a participar da pesquisa: **Adaptação cultural e validação do instrumento *WHO Surgical Safety Checklist: for Radiological Interventions ONLY*: versão para o português brasileiro.** O objetivo desta pesquisa é traduzir e tornar válido para os brasileiros uma lista de verificações, elaborada na Inglaterra, que ajuda os profissionais que atuam no setor de hemodinâmica a conferirem itens importantes que podem auxiliar na segurança e melhorar a prática clínica. Sua participação é importante, pois através dela poderemos verificar se uma lista de verificações pode auxiliar na segurança, prática clínica e na comunicação da equipe em procedimentos radiológicos invasivos. O estudo justifica-se visto que não há publicação sobre a implantação de uma lista de verificações de segurança no serviço de hemodinâmica na literatura brasileira.

Caso você aceite participar desta pesquisa será necessário a sua participação em dois momentos: 1 - comparar a versão do instrumento original com a versão traduzida e emitir seu parecer quanto às equivalências semântica, idiomática, cultural e conceitual, ao qual será enviado por e-mail. Com tempo estimado de 2 horas. 2 - participar de uma reunião consensual via Skype® para discussão dos aspectos polêmicos/controversos a fim de se obter uma única versão traduzida para o português, no local a ser definido. Com tempo estimado de 2 horas.

Não será feito nenhum procedimento que lhe traga qualquer desconforto ou risco.

Você não terá benefícios diretos. No entanto, a pesquisa poderá trazer informações importantes para auxiliar na segurança aos pacientes submetidos a procedimentos radiológicos invasivos.

Você poderá obter quaisquer informações relacionadas a sua participação nesta pesquisa, a qualquer momento que desejar, por meio dos pesquisadores do estudo. Sua participação é voluntária, e em decorrência dela você não receberá qualquer valor em dinheiro. Você não terá nenhum gasto por participar nesse estudo, pois qualquer gasto que você tenha por causa dessa pesquisa lhe será ressarcido. Você poderá não participar do estudo, ou se retirar a qualquer momento, sem que haja qualquer constrangimento junto aos pesquisadores, ou prejuízo quanto a sua rotina de trabalho, cargo ou função na instituição, bastando você dizer ao pesquisador que lhe entregou este documento. Você não será identificado neste estudo, pois a sua identidade será de conhecimento apenas dos pesquisadores da pesquisa, sendo garantido o seu sigilo e privacidade. Você tem direito a requerer indenização diante de eventuais danos que você sofra em decorrência dessa pesquisa.

Rubrica do participante	Data	Rubrica do pesquisador	Data

<u>Este documento deverá ser emitido em duas vias, uma para o participante e outra para o pesquisador.</u>

Universidade Federal do Triângulo Mineiro
Pós-graduação *Stricto Sensu* em Atenção à Saúde
Av. Getúlio Guaritá, 159 – Nossa Sra. da Abadia, Uberaba – MG, 38025-440

Contato dos pesquisadores:
Pesquisador(es):
Nome: Maria Helena Barbosa
E-mail: mhelena331@hotmail.com
Telefone: (34) 9.9166-9447
Endereço: Av. Getúlio Guaritá nº 107 - Bairro Abadia

Nome: Flavia Ana Pacheco
E-mail: Flavia.ana.pacheco@gmail.com
Telefone: (34) 9.9133-0609
Endereço: Av. Getúlio Guaritá nº 107 - Bairro Abadia

Em caso de dúvida em relação a esse documento, favor entrar em contato com o Comitê de Ética em Pesquisa da Universidade Federal do Triângulo Mineiro, pelo telefone (34) 3700-6776, ou no endereço Rua Madre Maria José, 122, Bairro Nossa Senhora da Abadia – Uberaba – MG – de segunda a sexta-feira, das 08:00 às 11:30 e das 13:00 às 17:30. Os Comitês de Ética em Pesquisa são colegiados criados para defender os interesses dos participantes de pesquisas, quanto a sua integridade e dignidade, e contribuir no desenvolvimento das pesquisas dentro dos padrões éticos.

**Rubrica do participante**	**Data**	**Rubrica do pesquisador**	**Data**

Este documento deverá ser emitido em duas vias, uma para o participante e outra para o pesquisador.

Universidade Federal do Triângulo Mineiro
Pós-graduação *Stricto Sensu* em Atenção à Saúde
Av. Getúlio Guaritá, 159 – Nossa Sra. da Abadia, Uberaba – MG, 38025-440

TERMO DE CONSENTIMENTO LIVRE, APÓS ESCLARECIMENTO

TÍTULO DA PESQUISA: **Adaptação cultural e validação do instrumento *WHO Surgical Safety Checklist: for Radiological Interventions ONLY*: versão para o português brasileiro**

Eu, ____, li e/ou ouvi o esclarecimento acima e compreendi para que serve o estudo e a quais procedimentos serei submetido. A explicação que recebi esclarece os riscos e benefícios do estudo. Eu entendi que sou livre para interromper minha participação a qualquer momento, sem justificar minha decisão e que isso não causará prejuízo quanto a minha rotina de trabalho, cargo ou função na instituição. Sei que meu nome não será divulgado, que não terei despesas e não receberei dinheiro para participar do estudo. Concordo em participar do estudo, **Adaptação cultural e validação do instrumento *WHO Surgical Safety Checklist: for Radiological Interventions ONLY*: versão para o português brasileiro,** e receberei uma via assinada deste documento.

Uberaba, .../ ..../...............

____
Assinatura do voluntário

________________________________ ________________________________

Assinatura do pesquisador responsável Assinatura do pesquisador assistente

Telefone de contato dos pesquisadores:

Flavia Ana Pacheco (34) 9.9133-0609

Rubrica do participante	Data	Rubrica do pesquisador	Data

Este documento deverá ser emitido em duas vias, uma para o participante e outra para o pesquisador.

APÊNDICE B – Carta-convite para composição do comitê de juízes.

Universidade Federal do Triângulo Mineiro
Programa de Pós-Graduação *Stricto Sensu* em Atenção à Saúde-PPGAS
Endereço: Av. Getúlio Guaritá, nº 107, Bairro Abadia, CEP 38.025-440, Uberaba – MG
Fone: (34) 3700-6607 - E-mail: sec.ppgas@uftm.edu.br

CARTA CONVITE PARA COMPOSIÇÃO DO COMITÊ DE JUÍZES

Prezado (a) Prof (a) Dr (a) ______________________________________,

Eu, Flávia Ana Pacheco, enfermeira, mestranda do Programa de Pós-Graduação em Atenção à Saúde da Universidade Federal do Triângulo Mineiro, sob a orientação da Profª Drª Maria Helena Barbosa, estou desenvolvendo uma pesquisa que objetiva realizar a adaptação cultural do instrumento ***WHO Surgical Safety Checklist: for Radiological Interventions ONLY*** e testar a validade de conteúdo e a confiabilidade interobservadores da versão adaptada do instrumento na versão para o português brasileiro. Tal instrumento foi desenvolvido pela *National Pacient Safety* em parceria com a *Royal College of Radiologists*, sendo este baseado no *checklist* para Segurança do Paciente Cirúrgico da *World Health Organization* (WHO) de 2008. Ressalto que o instrumento é livre para tradução e adaptação.

Considerando sua vasta experiência profissional e conhecimento científico venho por meio desta convidá-lo a ser um dos juízes que farão parte do Comitê de Juízes da pesquisa em questão.

Para tal, solicito a sua participação, na qualidade de juiz, com o propósito de avaliar as equivalências cultural, conceitual, semântica e idiomática do instrumento, bem como a validade de face de conteúdo, a qual analisa o conteúdo do instrumento, ou seja, será verificado se os itens constituem representativamente aquilo que se propõem avaliar.

Para alcance dos objetivos da pesquisa foi adotado o processo de adaptação cultural proposto por * Ferrer *et al.* (1996), o qual é dividido em seis partes: 1) Tradução de ***WHO Surgical Safety Checklist: for Radiological Interventions ONLY*** para português brasileiro; 2) Síntese - obtenção do primeiro consenso da versão em português; 3) Avaliação pelo comitê de juízes; 4) Retro-tradução; 5) Obtenção do consenso das versões em inglês e comparação com a versão original; 6) Análise semântica dos itens e 7) Pré-teste.

Desta forma, gostaria de contar com sua valiosa participação no processo de validação, que será em três fases: 1) Aceite de participação; 2) Avaliação primária do instrumento e 3) Consenso das alterações sugeridas pelo comitê de juízes. Ressalto que todas as etapas serão realizadas por meio eletrônico, não havendo a necessidade de deslocamento.

Agradeço antecipadamente pela atenção e estou à disposição para quaisquer esclarecimentos.

Atenciosamente,

Flávia Ana Pacheco

Mestranda em Atenção à Saúde
Universidade Federal do Triângulo Mineiro - UFTM
+55 34 99133-0609

* FERRER, M. et al. Validity and reliability of the St George's Respiratory Questionnaire after adaptation to a different language and culture: the Spanish example. **The European respiratory journal**, v. 9, n. 6, p. 1160-1166, 1996. ISSN PMID: 8804932.

APÊNDICE C – Versões Português 1 e 2

**Item**	Versão Português 1	Versão Português 2
	Lista de verificação de segurança cirúrgica da OMS: somente para intervenções radiológicas	Lista de checagem de segurança cirúrgica da OMS: apenas para intervenções radiológicas
	(adaptada da lista de verificações cirúrgicas da OMS)	(adaptada da lista de checagem cirúrgica da OMS)
	SIGN IN (ler em voz alta)	ENTRADA (ler em voz alta)
	Antes da indução da anestesia (local ou geral)	Antes de administrar a anestesia (local ou geral)
Item 1	Todos os membros da equipe se apresentaram indicando os seus nomes e funções?	Todos os membros da equipe se apresentaram com os seus nomes e funções?
	Sim	Sim
Item 2	Todos os membros da equipe confirmaram verbalmente:	Todos os membros da equipe confirmaram verbalmente:
	O nome do paciente?	O nome do paciente?
Item 3	O procedimento cirúrgico, o sítio cirúrgico e a posição planejados?	O procedimento cirúrgico, local cirúrgico e a posição planejada?
	No caso de anestesia geral, as duas perguntas acima devem ser colocadas no início da seção *TIME OUT*.	No caso de anestesia geral, as duas perguntas acima devem ser transferidas para o início da seção INTERVALO.

Item 4	O paciente confirmou sua identidade, o sítio cirúrgico, o procedimento e o consentimento?	O paciente confirmou sua identidade, local cirúrgico, procedimento e consentimento?
	Sim	Sim
Item 5	Os exames de imagem essenciais foram avaliados?	Os exames de imagem essenciais foram revisados?
	Sim	Sim
	Não se aplica	Não se aplica
Item 6	Todos os requisitos dos IRMER (regulamentos das radiações ionizantes nas exposições médicas) foram atendidos?	Todos os requisitos dos IRMER (regulamentos das radiações ionizantes nas exposições médicas) foram atendidos?
	Sim	Sim
Item 7	O sítio cirúrgico foi demarcado?	O local cirúrgico foi demarcado?
	Sim	Sim
	Não se aplica	Não se aplica
Item 8	Foi concluída a verificação da máquina de anestesia, dos equipamentos de monitoramento e da medicação?	A checagem da máquina de anestesia/equipamentos de monitoramento e medicação foi feita?
	Sim	Sim
	Não se aplica	Não se aplica
Item 9	O paciente possui:	O paciente possui:
	alergia conhecida?	alergia conhecida?

	Não	Não
	Sim	Sim
Item 10	risco previsto de perda sanguínea >500mL (7mL/kg em crianças)?	risco previsto de perda de sangue >500mL (7mL/kg em crianças)?
	Não	Não
	Sim (e acesso endovenoso /planejamento para fluidos adequados)	Sim (e acesso adequado endovenoso/para fluidos planejado)
Item 11	Os fatores de risco para hemorragia e insuficiência renal foram verificados?	Os fatores de risco para hemorragia e insuficiência renal foram verificados?
	Sim	Sim
	Não se aplica	Não se aplica
Item 12	Foi administrada profilaxia antibiótica?	Foi administrada profilaxia antibiótica?
	Sim	Sim
	Não se aplica	Não se aplica
Item 13	Foi administrada profilaxia tromboembólica?	Foi administrada profilaxia tromboembólica venosa?
	Sim	Sim
	Não se aplica	Não se aplica
Item 14	Os equipamentos necessários estão disponíveis e atualizados?	Os equipamentos necessários estão disponíveis e atualizados?

	Sim	Sim
Item 15	Existe algum procedimento crítico ou inesperado que você deseja comunicar à equipe?	Há alguma medida crítica ou inesperada que você queira informar à equipe?
	Sim	Sim
	Não se aplica	Não se aplica
	Somente no caso de anestesia geral	SOMENTE NO CASO DE ANESTESIA GERAL
	TIME OUT (ler em voz alta)	INTERVALO (ler em voz alta)
	Antes de iniciar a intervenção radiológica (por exemplo, agulha na pele)	Antes de iniciar a intervenção radiológica (por exemplo, agulha na pele)
	Eventos críticos previstos	Eventos críticos previstos
	Anestesista (se houver):	Anestesista (se presente):
Item 16	Foi concluída a verificação da máquina de anestesia?	A checagem da máquina de anestesia foi feita?
Item 17	O paciente apresenta risco de via aérea difícil/aspiração?	O paciente apresenta risco de aspiração/via aérea difícil?
	Sim	Sim
	Não se aplica	Não se aplica
Item 18	Existe alguma preocupação específica em relação ao paciente?	Há alguma preocupação específica quanto ao paciente?

Item 19	Qual a classificação ASA do paciente?	Qual a classificação ASA do paciente?
Item 20	Quais equipamentos de monitoramento e outros níveis específicos de suporte são necessários, por exemplo, para sangue?	Quais equipamentos de monitoramento e outros níveis específicos de suporte são necessários, por exemplo sangue?
	Médico e equipe de enfermagem:	Médico e equipe de enfermagem:
Item 21	Existe alguma questão ou preocupação relacionada aos equipamentos?	Há alguma questão ou preocupação relacionada aos equipamentos?
Item 22	Os procedimentos para evitar infecções no sítio cirúrgico (ISC) foram realizados?	O pacote de medidas de prevenção de infecções no sítio cirúrgico (ISC) foi aplicado?
	Sim	Sim
	Não se aplica	Não se aplica
	· Profilaxia antibiótica	· Profilaxia antibiótica
	· Alerta ao paciente	· Aviso ao paciente
	· Remoção de pelos	· Remoção de pelos
	· Controle glicêmico	· Controle glicêmico
Enunciado	*SIGN OUT* (ler em voz alta)	SAÍDA (ler em voz alta)
	Antes que algum membro da equipe saia da sala	Antes que algum membro da equipe saia da sala

	O médico e a equipe de enfermagem confirmaram verbalmente com a equipe:	O médico e a equipe de enfermagem confirmaram verbalmente com a equipe:
Item 23	Foram registrados o nome do procedimento e o lado onde foi realizado?	O nome do procedimento e o lado onde foi realizado foram registrados?
Item 24	Todos os instrumentais e agulhas utilizados foram relatados?	Todos os equipamentos invasivos utilizados foram discriminados?
Item 25	Foi registrado algum dispositivo implantado?	Foi registrado algum dispositivo implantado?
Item 26	As amostras colhidas foram rotuladas (incluindo o nome do paciente)?	As amostras colhidas foram rotuladas (incluindo o nome do paciente)?
Item 27	Foi identificado algum problema com equipamento para ser resolvido?	Foi identificado algum problema com equipamento que precisa ser resolvido?
	O radiologista, o anestesista e a equipe de enfermagem:	O radiologista, o anestesista e a equipe de enfermagem:
Item 28	Revisaram as instruções de cuidado pós-procedimento para este paciente?	Concordaram quanto as instruções de cuidado pós-procedimento para este paciente?
	Lembre-se de confirmar no sistema CRIS ou registrar que a lista de verificações foi realizada.	Lembre-se de escanear no sistema CRIS ou registrar se a lista de checagem foi concluída.
	Informações sobre o paciente	DETALHES DO PACIENTE
	Sobrenome:	Sobrenome:
	Nome:	Nome:

	Data de nascimento:	Data de nascimento:
	Número do sistema de saúde*:	Número NHS*:
	Data do procedimento:	Data do procedimento:
	*Se não houver número do sistema de saúde disponível no momento, um número temporário deve ser usado até obter o número definitivo.	*Se o número NHS não estiver disponível no momento, um número temporário deve ser utilizado até que se tenha a informação.
	A lista de verificações é somente para intervenções radiológicas.	A lista de checagem é apenas para intervenções radiológicas.
	Esta lista de verificações modificada não deve ser usada em outros procedimentos cirúrgicos.	Esta lista de checagem modificada não deve ser utilizada para outros procedimentos cirúrgicos.
	National Patient Safety Agency	Agência Nacional de Segurança do Paciente
	National Reporting and Learning Service	Serviço Nacional de Aprendizagem e Relato
	©*National Patient Safety Agency* 2010. Os direitos autorais e outros direitos de propriedade intelectual deste material pertencem à NPSA e todos os direitos são reservados. A NPSA autoriza as organizações de saúde do Reino Unido a reproduzir este material para uso educativo e não comercial.	©*National Patient Safety Agency* 2010. Os direitos autorais e outros direitos de propriedade intelectual deste material pertencem à NPSA e todos os direitos são reservados. A NPSA autoriza as organizações de saúde do Reino Unido a reproduzirem este material para uso educacional e não comercial.

APÊNDICE D – Versão Português Consenso 1

Lista de Verificação de Segurança Cirúrgica da OMS:
SOMENTE para intervenções radiológicas
(adaptada da Lista de Verificações Cirúrgicas da OMS)

THE ROYAL COLLEGE OF RADIOLOGISTS

NHS
National Patient Safety Agency
National Reporting and Learning Service

SIGN IN / ENTRADA (ler em voz alta)

Antes da indução anestésica (local ou geral)

Todos os membros da equipe se apresentaram com os seus nomes e funções?
☐ Sim

Todos os membros da equipe confirmaram verbalmente:
☐ O nome do paciente?
☐ O procedimento cirúrgico, o sítio cirúrgico e a posição planejedos?

No caso de anestesia geral, as duas perguntas acima devem ser colocadas no início da seção TIME OUT / INVTERVALO.

O paciente confirmou sua identidade, o sítio cirúrgico, o procedimento e o consentimento? ☐ Sim

Os exames de imagem essenciais foram revisados?
☐ Sim ☐ Não se aplica

Todos os requisitos dos IRMER (regulamentos das radiações ionizantes nas exposições médicas) foram atendidos? ☐ Sim

O sítio cirúrgico foi demarcado?
☐ Sim ☐ Não se aplica

A checagem do aparelho de anestesia/equipamentos de monitoramento e medicação foi feita?
☐ Sim ☐ Não se aplica

O paciente possui:
alergia conhecida?
☐ Não ☐ Sim
risco previsto de perda sanguínea >500ml (7ml/kg em crianças)?
☐ Não ☐ Sim (acesso endovenoso adequado/fluidos planejados)

Os fatores de risco para hemorragia e insuficiência renal foram verificados? ☐ Sim ☐ Não se aplica

Foi administrada profilaxia antibiótica?
☐ Sim ☐ Não se aplica
Foi administrada profilaxia tromboembólica venosa?
☐ Sim ☐ Não se aplica

Os equipamentos necessários estão disponíveis e dentro da validade?
☐ Sim

Existe algum procedimento crítico ou inesperado que você deseja comunicar à equipe?
☐ Sim ☐ Não se aplica

SOMENTE NO CASO DE ANESTESIA GERAL

TIME OUT/ INTERVALO (ler em voz alta)

Antes de iniciar a intervenção radiológica
(por exemplo, punção)

Eventos críticos previstos

Anestesia (se houver):
☐ A checagem do aparelho de anestesia foi realizada?

O paciente apresenta risco de aspiração/via aérea difícil?
☐ Sim ☐ Não se aplica

☐ Existe alguma preocupação específica em relação ao paciente?
☐ Qual a classificação ASA do paciente?
☐ Quais equipamentos de monitoramento e outros níveis específicos de suporte são necessários, por exemplo sangue?

Médico e equipe de enfermagem:
☐ Existe alguma questão ou preocupação relacionada aos equipamentos?

Os procedimentos para evitar infecções no sítio cirúrgico (ISC) foram realizados?
☐ Sim ☐ Não se aplica

- Profilaxia antibiótica
- Alerta ao paciente
- Remoção de pelos
- Controle glicêmico

A lista de verificações é somente para intervenções radiológicas

Esta lista de verificações modificada não deve ser usada em outros procedimentos cirúrgicos.

© National Patient Safety Agency 2010. Os direitos autorais e outros direitos de propriedade intelectual deste material pertencem à NPSA e todos os direitos são reservados. A NPSA autoriza as organizações de saúde do Reino Unido a reproduzir este material para uso educativo e não comercial.

SIGN OUT / SAÍDA (ler em voz alta)

Antes que algum membro da equipe saia da sala

O médico e a equipe de enfermagem confirmaram verbalmente com a equipe:
☐ O nome do procedimento e o lado onde foi realizado foram registrados?
☐ Todos os instrumentais e agulhas utilizados foram contabilizados?
☐ Foi registrado algum dispositivo implantado?
☐ As amostras colhidas foram rotuladas (incluindo o nome do paciente)?
☐ Foi identificado algum problema com equipamento que precisa ser resolvido?

O radiologista, o anestesista e a equipe de enfermagem:
☐ Concordaram quanto as instruções de cuidado pós-procedimento para este paciente?

Lembre-se de registrar no prontuário que a lista de verificações foi realizada

INFORMAÇÕES SOBRE O PACIENTE

Sobrenome:
Nome:
Data de nascimento:
Número de registro:
Data do procedimento:

*Se o número de registro não estiver disponível no momento, um número temporário deve ser utilizado até que se tenha a informação

www.nrls.npsa.nhs.uk

Ref: 1121 March 2010

APÊNDICE E – Versão em Português Consenso 2

WHO Surgical Safety Checklist: for Radiological Interventions ONLY

(adapted from the WHO Surgical Safety Checklist)

Versão Português-Brasileiro

THE ROYAL COLLEGE OF RADIOLOGISTS

NHS
National Patient Safety Agency
National Reporting and Learning Service

SIGN IN / ENTRADA (Ler em voz alta)

Antes da indução anestésica
(local, regional ou geral)

Todos os membros da equipe se apresentaram com os seus nomes e funções? ☐ Sim

Todos os membros da equipe confirmaram verbalmente:
☐ Qual o nome do paciente?
☐ Estão planejados o procedimento, o sítio cirúrgico e a posição?

No caso de anestesia geral, as duas perguntas acima devem ser colocadas no início da seção TIME OUT / INVTERVALO.

Estão confirmados: a identidade do paciente, o tipo e o local do procedimento e se há consentimento? ☐ Sim

Os exames de imagem essenciais foram revisados?
☐ Sim ☐ Não se aplica

Todos os requisitos dos IRMER (regulamentos das radiações ionizantes nas exposições médicas) foram atendidos? ☐ Sim

O sítio cirúrgico foi demarcado?
☐ Sim ☐ Não se aplica

A checagem do aparelho de anestesia/equipamentos de monitoramento e medicação foi realizada?
☐ Sim ☐ Não se aplica

O paciente possui:
alergia conhecida?
☐ Não ☐ Sim
há risco de perda sanguínea >500 ml (7 ml/kg em crianças)?
☐ Não ☐ Sim (acesso venoso adequado/fluidos planejados)

Os fatores de risco para hemorragia e insuficiência renal foram verificados? ☐ Sim ☐ Não se aplica

Foi administrada profilaxia antibiótica?
☐ Sim ☐ Não se aplica
Foi realizada profilaxia para tromboembolismo venoso?
☐ Sim ☐ Não se aplica

Os materiais necessários estão disponíveis e dentro da validade?
☐ Sim

Existe alguma etapa crítica ou inesperada que você deseja comunicar à equipe
☐ Sim ☐ Não se aplica

SOMENTE NO CASO DE ANESTESIA GERAL

TIME OUT / INTERVALO (Ler em voz alta)

Antes de iniciar a intervenção radiológica
(por exemplo, punção)

Eventos críticos previstos

Anestesista (se presente):
☐ A checagem do aparelho de anestesia foi realizada?

O paciente apresenta risco de aspiração/via aérea difícil?
☐ Sim ☐ Não se aplica

☐ Existe alguma preocupação específica em relação ao paciente?
☐ Qual a classificação da ASA do paciente?
☐ Quais equipamentos de monitoramento e outros tipos de suporte são necessários, por exemplo sangue?

Médico e equipe de enfermagem:
☐ Existe alguma preocupação relacionada aos equipamentos?

Os procedimentos para evitar infecções no sítio cirúrgico (ISC) foram realizados?
☐ Sim ☐ Não se aplica

- Profilaxia antibiótica
- Alerta ao paciente
- Remoção de pelos
- Controle glicêmico

O Checklist é somente para procedimentos radiológicos

Este Checklist não deve ser utilizado em outros procedimentos cirúrgicos.

SIGN OUT / SAÍDA (Ler em voz alta)

Antes que algum membro da equipe saia da sala

O médico e a equipe de enfermagem confirmam verbalmente com a equipe:
☐ O nome do procedimento e o lado onde foi realizado foram registrados?
☐ Todos os instrumentais e agulhas utilizados foram contabilizados?
☐ Foi registrado algum dispositivo implantado?
☐ As amostras colhidas foram rotuladas (incluindo o nome do paciente)?
☐ Foi identificado algum problema com equipamento que precisa ser resolvido?

O radiologista, o anestesista e a equipe de enfermagem:
☐ Concordaram quanto as instruções de cuidado pós-procedimento para este paciente?

Lembre-se de registrar no prontuário que o checklist foi realizado

INFORMAÇÕES SOBRE O PACIENTE

Sobrenome:
Nome:
Data de nascimento:
Número de registro:
Data do procedimento:

*Se o número de registro não estiver disponível no momento, um número temporário deve ser utilizado até que se tenha a informação

www.nrls.npsa.nhs.uk

© National Patient Safety Agency 2010. Os direitos autorais e outros direitos de propriedade intelectual deste material pertencem à NPSA e todos os direitos são reservados. A NPSA autoriza as organizações de saúde do Reino Unido a reproduzir este material para uso educativo e não comercial.

Ref: 1121 March 2010

APÊNDICE F – Versões Inglês 1 e 2.

**Item**	**Retrotradução 1**	**Retrotradução 2**
	WHO Surgical Safety Checklist: for Radiological Interventions ONLY	*WHO Surgical Safety Checklist: For Radiological Interventions ONLY*
	(adapted from the WHO Surgical Safety Checklist)	*(adapted from the WHO Surgical Safety Checklist) Portuguese-Brazilian version*
	SIGN IN (Read in a loud voice)	*SIGN IN (Read out loud)*
	Before anaesthetic induction (local, regional or general)	*Before induction of anesthesia (local, regional or general)*
Item 1	*Did all team members introduce themselves with their names and functions?*	*Have all the team members introduced themselves by name and function?*
	Yes	*Yes*
Item 2	*Did all team members verbally confirm:*	*All the team members verbally confirmed:*
	What is the patient's name?	*What is the patient’s name?*
Item 3	*Are the procedure, the surgical site, and the position planned?*	*Have the procedure, site and position been planned?*
	In the case of general anaesthesia, the two questions above should be asked at the beginning of the TIME OUT section.	*In the case of general anesthesia, the two questions above should be placed at the start of the TIME OUT section.*

Item 4	*Are the patient's identity, the type and location of the procedure, and the consent confirmed?*	*Has the patient’s identity, type and site of the procedure and consent been confirmed?*
	Yes	*Yes*
Item 5	*Have essential imaging exams been reviewed?*	*Have all the necessary imaging tests been reviewed?*
	Yes	*Yes*
	Not applicable	*Not applicable*
Item 6	*Have all requirements of the IRMER (ionizing radiation regulations on medical exposures) been met?*	*Have all the IRMER requirements (Ionizing Radiation (Medical Exposure) Regulations) been met?*
	Yes	*Yes*
Item 7	*Has the surgical site been demarcated?*	*Has the site been marked?*
	Yes	*Yes*
	Not applicable	*Not applicable*
Item 8	*Has the anaesthesia device/monitoring and medication equipment check been performed?*	*Has the anesthesia machine/monitoring and measurement equipment been checked?*
	Yes	*Yes*
	Not applicable	*Not applicable*
Item 9	*Does the patient have:*	*Does the patient have:*
	A known allergy?	*a known allergy?*

	No	*No*
	Yes	*Yes*
Item 10	*Have the risk factors for bleeding and renal failure been verified?*	*Is there any risk of >500mL blood loss (7mL/kg in children)?*
	No	*No*
	Yes (adequate venous access/planned fluids)	*Yes (adequate venous access/fluids planned)*
Item 11	*Have the risk factors for bleeding and renal failure been verified?*	*Have the risk factors for hemorrhaging and kidney failure been checked?*
	Yes	*Yes*
	Not applicable	*Not applicable*
Item 12	*Has antibiotic prophylaxis been administered?*	*Has antibiotic prophylaxis been given?*
	Yes	*Yes*
	Not applicable	*Not applicable*
Item 13	*Has prophylaxis been performed for venous thromboembolism?*	*Has venous thromboembolism prophylaxis been performed?*
	Yes	*Yes*
	Not applicable	*Not applicable*
Item 14	*Are the necessary materials available and within their validity?*	*Are all the necessary materials available and within the expiration date?*

	Yes	*Yes*
Item 15	*Are there any critical or unexpected steps you want to communicate to the team?*	*Is there any critical or unanticipated stage you want to communicate to the team?*
	Yes	*Yes*
	Not applicable	*Not applicable*
	ONLY IN THE CASE OF GENERAL ANAESTHESIA	*ONLY IN THE CASE OF GENERAL ANESTHESIA*
	TIME OUT (Read in a loud voice)	*TIME OUT (Read out loud)*
	Before starting radiological intervention (e.g. puncture)	*Before starting the radiological intervention (for example, puncture)*
	Predicted critical events	*Anticipated critical events*
	Anaesthetist (if present):	*Anesthetist (if present):*
Item 16	*Was an anaesthesia device check performed?*	*Has the anesthesia machine been checked?*
Item 17	*Is the patient at risk for aspiration/difficult airway?*	*Does the patient have any aspiration/difficult airway risk?*
	Yes	*Yes*
	Not applicable	*Not applicable*
Item 18	*Is there any specific concern regarding the patient?*	*Are there any specific patient-related concerns?*
Item 19	*What is the patient's ASA classification?*	*What is the patient’s ASA classification?*

Item 20	*What monitoring equipment and other support is needed, for example blood?*	*What monitoring equipment and other types of support are necessary, such as blood?*
	Doctor and Nursing Team:	*Physician and nursing team:*
Item 21	*Are there any equipment concerns?*	*Are there equipment-related concerns?*
Item 22	*Have procedures to prevent surgical site infections (SSI) been performed?*	*Were the procedures to avoid surgical site infections (SSI) performed?*
	Yes	*Yes*
	Not applicable	*Not applicable*
	Antibiotic Prophylaxis	*Antibiotic prophylaxis*
	Patient Alert	*Patient alert*
	Body hair Removal	*Removal of hair*
	Glycemic Control	*Glycemic control*
Enunciado	*SIGN OUT (Read in a loud voice)*	*SIGN OUT (Read out loud)*
	Before any staff member leaves the room	*Before any team member leaves the room*
	The doctor and the nursing team verbally confirm with the team:	*The physician and nursing team verbally confirm with the team:*
Item 23	*Have the name of the procedure and the site where it was performed been recorded?*	*Has the name of the procedure and the side where it was performed been recorded?*

Item 24	*Have all instruments and needles used been accounted for?*	*Were all the instruments and needles used counted?*
Item 25	*Have any implanted devices been registered?*	*Was any implanted device recorded?*
Item 26	*Have the samples taken been labelled (including the patient's name)?*	*Were the samples taken labeled (including the patient's name)?*
Item 27	*Has a problem been identified with equipment that needs to be resolved?*	*Were there any problems detected with the equipment that need to be resolved?*
	Radiologist, anaesthetist and nursing team:	*The radiologist, anesthetist and nursing team:*
Item 28	*Did the team agree with the post-procedural care instructions for this patient?*	*Did they agree with the post-procedure care instructions for this patient?*
	Remember to record in the chart that the checklist was performed	*Remember to record on the patient's medical record that the checklist was completed*
	PATIENT INFORMATION	*PATIENT INFORMATION*
	Last Name:	*Surname:*
	Name:	*Name:*
	Date of birth:	*Date of birth:*
	Registration number:	*Registration number:*
	Date of the procedure:	*Date of the procedure:*

	** If the registration number is not currently available, a temporary number should be used until you have the information*	**If the registration number is not available at the time, a temporary number should be used until this information is obtained.*
	Checklist for Radiological Procedures Only	*The checklist is only for radiological procedures*
	This Checklist should not be used in other surgical procedures.	*This checklist should not be used in other surgical procedures.*
		National Patient Safety Agency 2010. The copyrights and other intellectual property rights of this material belong to the NPSA and all rights are reserved. The NPSA authorizes health organizations in the United Kingdom to reproduce this material for educational and non-commercial purposes.

APÊNDICE G – Versão Pré-Final.

WHO Surgical Safety Checklist: for Radiological Interventions ONLY

(adapted from the WHO Surgical Safety Checklist)
Versão Português-Brasileiro

THE ROYAL COLLEGE OF RADIOLOGISTS

NHS
National Patient Safety Agency
National Reporting and Learning Service

SIGN IN / ENTRADA (Ler em voz alta)

Antes da indução anestésica
(local, regional ou geral)

Todos os membros da equipe se apresentaram com os seus nomes e funções?
☐ Sim

Todos os membros da equipe confirmaram verbalmente:
☐ Qual o nome do paciente?
☐ Estão planejados o procedimento, o sítio cirúrgico e a posição?

No caso de anestesia geral, as duas perguntas acima devem ser colocadas no início da seção TIME OUT / INVTERVALO.

Estão confirmados: a identidade do paciente, o tipo e o local do procedimento e se há consentimento? ☐ Sim

Os exames de imagem essenciais foram revisados?
☐ Sim ☐ Não se aplica

Todos os requisitos dos IRMER (regulamentos das radiações ionizantes nas exposições médicas) foram atendidos? ☐ Sim

O sítio cirúrgico foi demarcado?
☐ Sim ☐ Não se aplica

A checagem do aparelho de anestesia/equipamentos de monitoramento e medicação foi realizada?
☐ Sim ☐ Não se aplica

O paciente possui:
alergia conhecida?
☐ Não ☐ Sim
há risco de perda sanguínea >500 ml (7 ml/kg em crianças)?
☐ Não ☐ Sim (acesso venoso adequado/fluidos planejados)

Os fatores de risco para hemorragia e insuficiência renal foram verificados? ☐ Sim ☐ Não se aplica

Foi administrada profilaxia antibiótica?
☐ Sim ☐ Não se aplica
Foi realizada profilaxia para tromboembolismo venoso?
☐ Sim ☐ Não se aplica

Os materiais necessários estão disponíveis e dentro da validade?
☐ Sim

Existe alguma etapa critica ou inesperada que você deseja comunicar à equipe
☐ Sim ☐ Não se aplica

SOMENTE NO CASO DE ANESTESIA GERAL

TIME OUT / INTERVALO (Ler em voz alta)

Antes de iniciar a intervenção radiológica
(por exemplo, punção)

Eventos críticos previstos

Anestesista (se presente):
☐ A checagem do aparelho de anestesia foi realizada?

O paciente apresenta risco de aspiração/via aérea difícil?
☐ Sim ☐ Não se aplica

☐ Existe alguma preocupação específica em relação ao paciente?
☐ Qual a classificação da ASA do paciente?
☐ Quais equipamentos de monitoramento e outros tipos de suporte são necessários, por exemplo sangue?

Médico e equipe de enfermagem:
☐ Existe alguma preocupação relacionada aos equipamentos?

Os procedimentos para evitar infecções no sítio cirúrgico (ISC) foram realizados?
☐ Sim ☐ Não se aplica

- Profilaxia antibiótica
- Alerta ao paciente
- Remoção de pelos
- Controle glicêmico

O Checklist é somente para procedimentos radiológicos

Este Checklist não deve ser utilizado em outros procedimentos cirúrgicos.

© National Patient Safety Agency 2010. Os direitos autorais e outros direitos de propriedade intelectual deste material pertencem à NPSA e todos os direitos são reservados. A NPSA autoriza as organizações de saúde do Reino Unido a reproduzir este material para uso educativo e não comercial.

SIGN OUT / SAÍDA (Ler em voz alta)

Antes que algum membro da equipe saia da sala

O médico e a equipe de enfermagem confirmam verbalmente com a equipe:
☐ O nome do procedimento e o lado onde foi realizado foram registrados?
☐ Todos os instrumentais e agulhas utilizados foram contabilizados?
☐ Foi registrado algum dispositivo implantado?
☐ As amostras colhidas foram rotuladas (incluindo o nome do paciente)?
☐ Foi identificado algum problema com equipamento que precisa ser resolvido?

O radiologista, o anestesista e a equipe de enfermagem:
☐ Concordaram quanto as instruções de cuidado pós-procedimento para este paciente?

Lembre-se de registrar no prontuário que o checklist foi realizado

INFORMAÇÕES SOBRE O PACIENTE

Sobrenome:
Nome:
Data de nascimento:
Número de registro:
Data do procedimento:

*Se o número de registro não estiver disponível no momento, um número temporário deve ser utilizado até que se tenha a informação

www.nrls.npsa.nhs.uk

Ref: 1121 March 2010

APÊNDICE H - Versão Final.

WHO Surgical Safety Checklist: for Radiological Interventions ONLY

(adapted from the WHO Surgical Safety Checklist)
Versão Português-Brasileiro

THE ROYAL COLLEGE OF RADIOLOGISTS

NHS
National Patient Safety Agency
National Reporting and Learning Service

SIGN IN / ENTRADA (Ler em voz alta)

Antes da indução anestésica
(local, regional ou geral)

Todos os membros da equipe se apresentaram com os seus nomes e funções? ☐ Sim

Todos os membros da equipe confirmaram verbalmente:
☐ Qual o nome do paciente?
☐ Estão planejados o procedimento, o sítio cirúrgico e a posição?

No caso de anestesia geral, as duas perguntas acima devem ser colocadas no início da seção TIME OUT / INVTERVALO.

O paciente confirmou sua identidade, o sítio cirúrgico, o procedimento e o consentimento? ☐ Sim

Os exames de imagem essenciais foram revisados?
☐ Sim ☐ Não se aplica

Todos os requisitos dos IRMER (regulamentos das radiações ionizantes nas exposições médicas) foram atendidos? ☐ Sim

O sítio cirúrgico foi demarcado?
☐ Sim ☐ Não se aplica

A checagem do aparelho de anestesia/equipamentos de monitoramento e medicação foi realizada?
☐ Sim ☐ Não se aplica

O paciente possui:
alergia conhecida?
☐ Não ☐ Sim
há risco de perda sanguínea >500 ml (7 ml/kg em crianças)?
☐ Não ☐ Sim (acesso venoso adequado/fluidos planejados)

Os fatores de risco para hemorragia e insuficiência renal foram verificados? ☐ Sim ☐ Não se aplica

Foi administrada profilaxia antibiótica?
☐ Sim ☐ Não se aplica
Foi realizada profilaxia para tromboembolismo venoso?
☐ Sim ☐ Não se aplica

Os materiais necessários estão disponíveis e dentro da validade?
☐ Sim

Existe alguma etapa crítica ou inesperada que você deseja comunicar à equipe
☐ Sim ☐ Não se aplica

SOMENTE NO CASO DE ANESTESIA GERAL

TIME OUT / INTERVALO (Ler em voz alta)

Antes de iniciar a intervenção radiológica
(por exemplo, punção)

Eventos críticos previstos

Anestesista (se presente):
☐ A checagem do aparelho de anestesia foi realizada?

O paciente apresenta risco de aspiração/via aérea difícil?
☐ Sim ☐ Não se aplica

☐ Existe alguma preocupação específica em relação ao paciente?
☐ Qual a classificação da ASA do paciente?
☐ Quais equipamentos de monitoramento e outros tipos de suporte são necessários, por exemplo sangue?

Médico e equipe de enfermagem:
☐ Existe alguma preocupação relacionada aos equipamentos?

Os procedimentos para evitar infecções no sítio cirúrgico (ISC) foram realizados?
☐ Sim ☐ Não se aplica

- Profilaxia antibiótica
- Alerta ao paciente
- Remoção de pelos
- Controle glicêmico

O Checklist é somente para procedimentos radiológicos

Este Checklist não deve ser utilizado em outros procedimentos cirúrgicos.

© National Patient Safety Agency 2010. Os direitos autorais e outros direitos de propriedade intelectual deste material pertencem à NPSA e todos os direitos são reservados. A NPSA autoriza as organizações de saúde do Reino Unido a reproduzir este material para uso educativo e não comercial.

SIGN OUT / SAÍDA (Ler em voz alta)

Antes que algum membro da equipe saia da sala

O médico e a equipe de enfermagem confirmam verbalmente com a equipe:
☐ O nome do procedimento e o lado onde foi realizado foram registrados?
☐ Todos os instrumentais e agulhas utilizados foram contabilizados?
☐ Foi registrado algum dispositivo implantado?
☐ As amostras colhidas foram rotuladas (incluindo o nome do paciente)?
☐ Foi identificado algum problema com equipamento que precisa ser resolvido?

O radiologista, o anestesista e a equipe de enfermagem:
☐ Concordaram quanto as instruções de cuidado pós-procedimento para este paciente?

Lembre-se de registrar no prontuário que o checklist foi realizado

INFORMAÇÕES SOBRE O PACIENTE

Sobrenome:
Nome:
Data de nascimento:
Número de registro:
Data do procedimento:

*Se o número de registro não estiver disponível no momento, um número temporário deve ser utilizado até que se tenha a informação

www.nrls.npsa.nhs.uk

Ref: 1121 March 2010
